# Near-saturated and complete genetic linkage map of black spruce (*Picea mariana*)

**DOI:** 10.1186/1471-2164-11-515

**Published:** 2010-09-24

**Authors:** Bum-Yong Kang, Ishminder K Mann, John E Major, Om P Rajora

**Affiliations:** 1 Forest Genetics and Biotechnology Group, Department of Biology, Life Sciences Centre, Dalhousie University, Halifax, NS, B3 H 4J1, Canada; 2 Current Address: Canada Research Chair in Forest and Conservation Genomics and Biotechnology, Canadian Genomics and Conservation Genetics Institute, Faculty of Forestry and Environmental Management, PO Box 44000, 28 Dineen Drive, University of New Brunswick, Fredericton, NB, E3B 5A3, Canada; 3 Natural Resources Canada, Canadian Forest Service - Atlantic Forestry Centre, PO Box 4000, 1350 Regent Street, Fredericton, NB, E3B 5P7, Canada; 4 Current Address: Division of Pulmonary, Allergy, and Critical Care Medicine, Emory University School of Medicine and Atlanta Veterans Affairs Medical Center, 1670 Clairmont Road, Decatur, GA 30033, USA

## Abstract

**Background:**

Genetic maps provide an important genomic resource for understanding genome organization and evolution, comparative genomics, mapping genes and quantitative trait loci, and associating genomic segments with phenotypic traits. Spruce (*Picea*) genomics work is quite challenging, mainly because of extremely large size and highly repetitive nature of its genome, unsequenced and poorly understood genome, and the general lack of advanced-generation pedigrees. Our goal was to construct a high-density genetic linkage map of black spruce (*Picea mariana*, 2n = 24), which is a predominant, transcontinental species of the North American boreal and temperate forests, with high ecological and economic importance.

**Results:**

We have developed a near-saturated and complete genetic linkage map of black spruce using a three-generation outbred pedigree and amplified fragment length polymorphism (AFLP), selectively amplified microsatellite polymorphic loci (SAMPL), expressed sequence tag polymorphism (ESTP), and microsatellite (mostly cDNA based) markers. Maternal, paternal, and consensus genetic linkage maps were constructed. The maternal, paternal, and consensus maps in our study consistently coalesced into 12 linkage groups, corresponding to the haploid chromosome number (1n = 1x = 12) of 12 in the genus *Picea*. The maternal map had 816 and the paternal map 743 markers distributed over 12 linkage groups each. The consensus map consisted of 1,111 markers distributed over 12 linkage groups, and covered almost the entire (> 97%) black spruce genome. The mapped markers included 809 AFLPs, 255 SAMPL, 42 microsatellites, and 5 ESTPs. Total estimated length of the genetic map was 1,770 cM, with an average of one marker every 1.6 cM. The maternal, paternal and consensus genetic maps aligned almost perfectly.

**Conclusion:**

We have constructed the first high density to near-saturated genetic linkage map of black spruce, with greater than 97% genome coverage. Also, this is the first genetic map based on a three-generation outbred pedigree in the genus *Picea*. The genome length in *P. mariana *is likely to be about 1,800 cM. The genetic maps developed in our study can serve as a reference map for various genomics studies and applications in *Picea a*nd Pinaceae.

## Background

Genetic maps provide an important genomic resource for understanding genome organization and evolution, comparative genomics, mapping genes and quantitative trait loci, and associating genes and genomic segments with phenotypic traits, especially in those species whose genomes are not yet completely sequenced. For understanding the genetic architecture of species, genetic maps with high levels of genome coverage and confidence in the marker order are required. High-density genetic maps and identification of genes or genetic factors controlling traits related to productivity, health, and adaptation to climatic change could accelerate forest tree improvement programs. Conifers are economically and ecologically important, and are the dominant tree species of the boreal and temperate forests. Genetic mapping and other genomics research is challenging in conifers, mainly because of their very large genome size (~25-30 Gbp) [1], the long time required to reach sexual maturity, inbreeding depression, and a general lack of advanced-generation pedigrees. The genome of any conifer species has yet to be completely sequenced.

*Picea *(spruce) is the second largest genus after *Pinus *(pine) in the family Pinaceae of conifers. Black spruce (*Picea mariana *(Mill.) B.S.P.) is a widespread transcontinental species of the North American boreal and temperate forests [2], and has great ecological and economic importance. It is one of the most important trees in Canada for the production of pulp and paper, and is one of the most important reforestation species in Canada [3]. Black spruce is a diploid species with haploid chromosome number (n) of 12 (2n = 2x = 24), like most other Pinaceae members. The estimated genome size of black spruce is large (1C = 15.8 pg; [4]; http://www.rbgkew.org.uk/cval/homepage.html) with an approximate 2C genome length of about 31,000 mpb http://www.rbgkew.org.uk/cval/homepage.html.

Although the first genetic linkage map in conifers was constructed for a single white spruce (*Picea glauca *(Moench) Voss) tree in 1992 from the analysis of haploid megagametophytes [5], the progress in the spruce genome mapping has been rather slow, particularly compared with the genus *Pinus*. Genetic linkage maps have been constructed for Norway spruce (*Picea abies *L.) [6-9], white spruce [5,10,11], and a black × red spruce (*Picea rubens *Sarg.) hybrid complex with an unknown proportion of the black spruce and red spruce genetic contribution to this hybrid [12,13]. A parentage test with species-specific DNA markers revealed that the crosses used in [12] and [13] harbored a substantial amount of the red spruce genetic background. The markers used in the above-reviewed genome mapping studies were random amplified polymorphic DNA (RAPD) or a combination of RAPD, amplified fragment length polymorphism (AFLP), microsatellite/simple sequence repeat (SSR), expressed sequence tag polymorphism (ESTP), selectively amplified microsatellite polymorphic loci (SAMPL), single nucleotide polymorphism (SNPs), and/or 5 S rDNA. With the exception of the maps constructed for Norway spruce and white spruce from *F*_1 _mapping populations [9,11] and the map constructed for black × red spruce hybrids from *F*_1 _and *BC*_1 _mapping populations [12,13], all other maps were constructed for single trees from the segregation of a small number of markers in haploid megagametophytes. Single-tree genetic maps are of limited value. In predominantly outcrossing plants, such as conifers, a three-generation outbred pedigree (TGOP) is considered to be more informative than *F*_1 _or *BC*_1 _pedigree [14]. However, there is no published genetic linkage map in the genus *Picea *based on a TGOP. Moreover, most of the published spruce genetic linkage maps have not coalesced into 12 linkage groups corresponding to the haploid chromosome number of *Picea*. The linkage groups have ranged from 12 to 29. The first single tree genetic linkage map of white spruce developed from 47 RAPD markers coalesced into 12 linkage groups. Although the consensus map of *Picea abies *[9] and the composite map of *P. mariana *X *P. rubens *hybrids [12,13] coalesced into 12 linkage groups, the maternal and/or paternal maps in these species coalesced into 13-23 linkage groups. There is no information published on a genetic linkage map in pure black spruce.

The objective of this study was to construct a high-density genetic linkage map of black spruce. We have used a three-generation outbred pedigree (TGOP) to develop a high density to near-saturated genetic map of black spruce. Here, we report maternal, paternal, and near-saturated consensus genetic linkage maps developed for black spruce using AFLP, SAMPL, ESTP, and SSR markers.

## Results

### AFLP markers

Forty AFLP primer combinations generated 809 markers segregating according to the expected Mendelian ratios. The number of polymorphic fragments ranged from 2 to 52, with an average of 20 polymorphic fragments per primer combination (Table 1). The average number of polymorphic fragments obtained per primer combination was 27, 18, 16 with the use of three, four, and five selective nucleotides (at the selective amplification step), respectively. The size of the segregating polymorphic fragments without adapters ranged from 40 to 660 bp, with only one mapped fragment below 50 bp and seven between 50 and 60 bp. Of the 809 markers, 485 segregated in ratio of 1:1 and 324 in ratio of 3:1. The number of markers segregating in the 1:1 ratio was 253 in the maternal parent and 232 in the paternal parent.

**Table 1:** AFLP primer combinations used, and the number and size of polymorphic fragments, and their segregation ratios.

Primer combinations	Size of fragments (bp)	Total number of polymorphic markers	Mendelian segregation	
			
			1:1	3:1
A-EAAC-MCCAC	54-340	52	31	21
A-EAAC-MCCACC	54-134	2	2	0
A-EAAC-MCCAG	65-502	17	17	0
A-EAAC-MCCATC	95-604	31	22	9
A-EAAG-MCCAG	80-236	18	11	7
A-EAAG-MCCATC	66-324	25	20	5
A-EACA-MCCAT	56-264	7	7	0
A-EACG-MCAT	52-198	22	14	8
A-EACG-MCCAG	40-245	14	8	6
A-EACG-MCTG	73-275	13	2	11
A-EACT-MCCAA	54-321	35	26	9
A-EACT-MCCTA	54-475	28	20	8
A-EAGC-MCTC	60-547	35	9	26
A-EAGC-MCTG	45-660	47	25	22
A-EACG-MCCAA	126-275	14	7	7
A-EACG-MCCTA	60-430	30	12	18
A-EACG-MCCGC	56-622	10	8	2
A-EACT-MCCAC	59-100	5	1	4
A-EACT-MCCAG	56-70	7	3	4
A-EACT-MCTA	74-327	32	19	13
A-EACT-MCAC	61-397	33	22	11
A-EACT-MCAT	80-262	27	15	12
A-EACG-MCCAT	66-148	8	4	4
A-EACG-MCCAC	67-327	11	4	7
A-EACG-MCTC	90-280	8	4	4
A-EACG-MCCAGC	78-210	12	11	1
A-EAAC-MCCTA	62-240	27	13	14
A-EAAC-MCCAT	69-414	23	13	10
A-EAAC-MCCAA	67-271	25	14	11
A-EAAC-MCCGA	66-194	11	6	5
A-EACA-MCAC	67-292	24	15	9
A-EACA-MCCACC	69-391	21	16	5
A-EACA-MCCGA	67-262	20	11	9
A-EACA-MCTA	60-261	33	26	7
A-EACA-MCCAC	67-178	9	7	2
A-EAAG-MCCAT	66-276	18	12	6
A-EAAG-MCCACC	65-121	9	5	4
A-EAAG-MCCAC	64-311	18	9	9
A-EAAG-MCCAA	69-249	11	7	4
A-EAAG-MCCTA	66-153	17	7	10

**Total**		**809**	**485**	**324**

### SAMPL markers

A total of 255 SAMPL markers, segregating according to the expected Mendelian ratios, was obtained from the 12 SAMPL-*Mse*I primer combinations (Table 2; Table 3). The fragment size without adapters ranged from 32 to 661 bp (Table 3). Only six mapped SAMPL markers were of < 50 bp and 6 from 50 to 60 bp without adapters. The number of polymorphic fragments ranged from 2 to 44, with an average of 21 polymorphic fragments per SAMPL primer combination. Of the 255 SAMPL markers, 149 segregated in the ratio of 1:1, whereas 106 SAMPL markers segregated in the ratio of 3:1. The number of SAMPL markers segregating in the 1:1 ratio was 94 in the maternal parent and 54 in the paternal parent.

**Table 2:** SAMPL primers developed from *Lactuca *(from Witsenboer et al. [41]) compound microsatellite repeats and used for SAMPL marker mapping in the black spruce mapping population.

Primer name	Primer sequence (5'→3')	Compound repeats
SL3	ACA CAC ACA CAC ACA TAT AA	A(CA)7 (TA)2A
SL4	TGT GTG TGT GTG TGT ATA	T (GT)7 (AT)2
SL5	CTC TCT CTC ACA CAC ACA CA	C(TC)4 (AC)4A
SL6	CTC TCT CTC GTG TGT GTG	C(TC)4 (GT)4G

**Table 3:** SAMPL primer and *Mse*I primer extension combinations used, and the number and size of polymorphic fragments, and their segregation ratios.

Primer combinations	DNA fragments size (bp)	Total number of polymorphic markers	Mendelian segregation	
			
			1:1	3:1
SL3-MCTT	91-315	20	17	3
SL3-MCAC	32-661	39	18	21
SL3-MCCG	40-416	6	6	0
SL4-MCTT	40-612	44	23	21
SL4-MCAC	57-440	23	11	12
SL4-MCCG	60-640	23	4	19
SL5-MCTT	88-223	7	7	0
SL5-MCAC	48-239	2	2	0
SL5-MCCG	50-437	13	12	1
SL6-MCTT	66-277	40	35	5
SL6-MCAC	66-272	18	4	14
SL6-MCCG	66-248	20	10	10

**Total**		**255**	**149**	**106**

### Microsatellite markers

Twenty white spruce EST-based SSR loci were polymorphic between the parents of the mapping population and were mapped on the consensus map (Table 4). Six of the 20 polymorphic loci were heterozygous in the female parent as dominant makers and fourteen in both parents as co-dominant makers. Two alleles at a SSR locus heterozygous in the male or female parent, segregated in a 1:1 ratio in the progeny. Where both the parents were heterozygous, the progeny segregated either in a 1:2:1 or 1:1:1:1 ratio for their parental alleles (2-4). The known genes mapped using the microsatellites from white spruce EST sequences were as follows: Linkage Group (LG) I - *Cytochrome B561 *(RPGSE40); LG III - *TIR/P-loop/LRR *(RPGSE37); LG VI - *Cytochrome P450 *(RPGSE10) and *putative UNC-50 *(RPGSE46); LG VII - *Early light inducible protein *(RPGSE11) and *Chloroplast nucleoid DNA binding protein *(RPGSE34); LG VIII - *Homeotic protein BEL1 *(RPGSE02); LG IX - *Auxin-induced protein 1 *(RPGSE13); and LG XI*- Putative Beta glycosidase *(RPGSE03) and *Gibberellin 12-oxidase *(RPGSE04).

**Table 4:** Microsatellite DNA markers used for genetic mapping in black spruce (*Picea mariana*).

Source	Number of markers mapped		
	
	Consensus map	Maternal map	Paternal map
*Picea glauca *ESTs	20	20	14
*Picea mariana *ESTs	6	4	2
*Picea mariana *genomic sequences	16	11	8

**Total**	**42**	**35**	**24**

Six black spruce EST-based SSR loci that were polymorphic between the parents as dominant markers were mapped on the consensus map; four were heterozygous in the female and two in the male parent for a null allele (Table 4). These markers segregated in a 1:1 ratio in the progeny. Sixteen black spruce genomic sequence-based SSR loci were polymorphic between the parents and were mapped on the consensus map (Table 4). Eight microsatellite markers were heterozygous for a null allele in the female parent and five were heterozygous in the male parent. These markers segregated in a 1:1 ratio as dominant markers. Three microsatellite loci were heterozygous in both parents and the progeny segregated in a 1:2:1 or 1:1:1:1 ratio as co-dominant markers.

### ESTP markers

**Figure 1:**
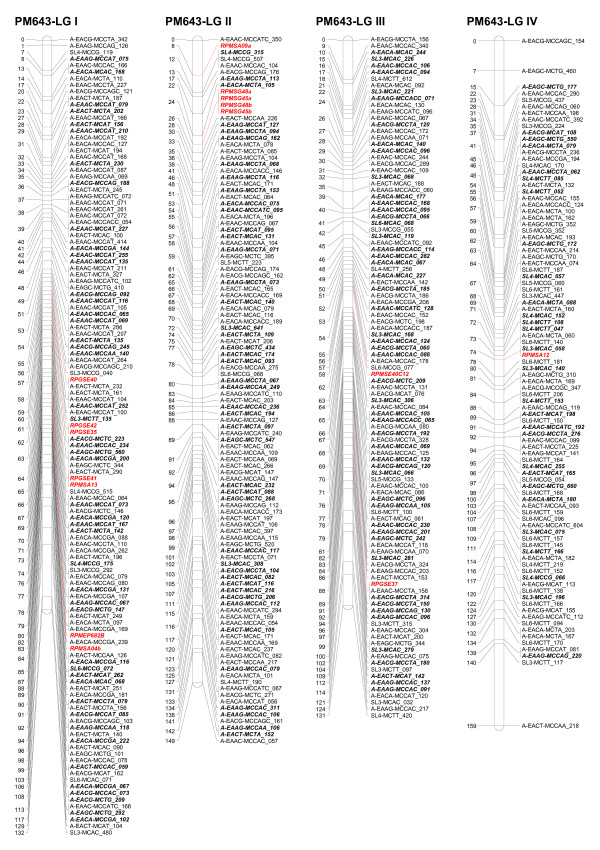
Linkage groups I to IV of the consensus genetic linkage map of black spruce (*Picea mariana*) constructed using 809 AFLPs (*A), 255 SAMPL (*S), 5 ESTPs (italicized and in red) and 42 SSR (italicized and in red color) markers. Names of the markers are provided on the right side of the linkage groups, with the DNA fragment size in bp. Genetic map distances, in cM, are provided on the left side of the linkage groups. AFLP and SAMPL markers in bold and italics are intercross markers.

**Figure 2:**
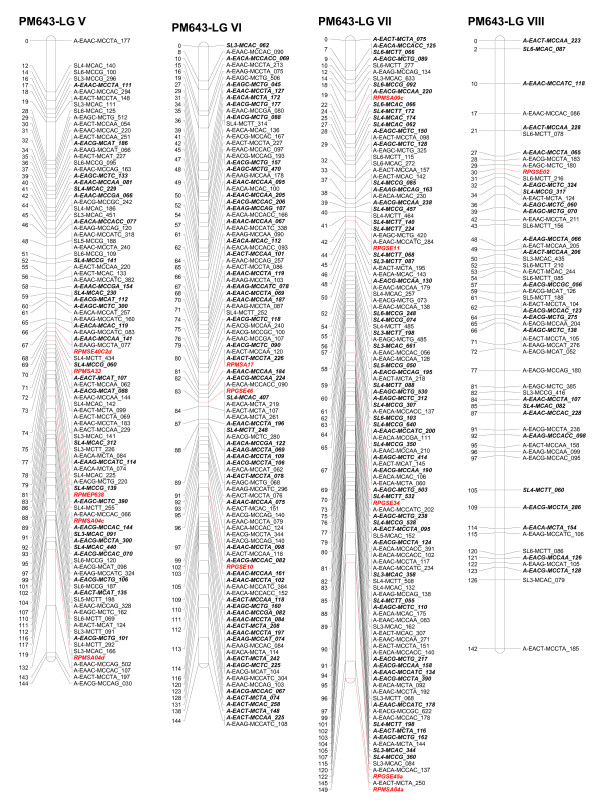
Linkage groups V to VIII of the consensus genetic linkage map of black spruce (*Picea mariana*) constructed using 809 AFLPs (*A), 255 SAMPL (*S), 5 ESTPs (italicized and in red) and 42 SSR (italicized and in red color) markers. Names of the markers are provided on the right side of the linkage groups, with the DNA fragment size in bp. Genetic map distances, in cM, are provided on the left side of the linkage groups. AFLP and SAMPL markers in bold and italics are intercross markers.

**Figure 3:**
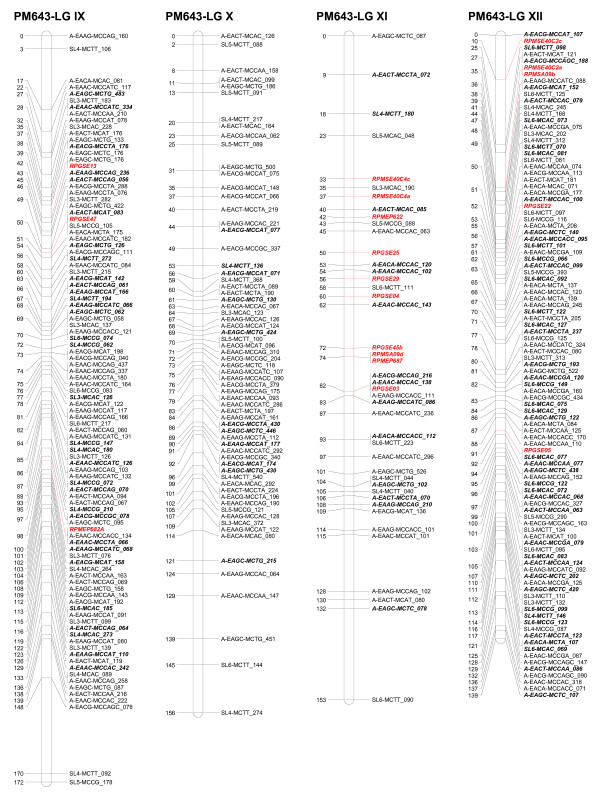
Linkage groups IX to XII of the consensus genetic linkage map of black spruce (*Picea mariana*) constructed using 809 AFLPs (*A), 255 SAMPL (*S), 5 ESTPs (italicized and in red) and 42 SSR (italicized and in red color) markers. Names of the markers are provided on the right side of the linkage groups, with the DNA fragment size in bp. Genetic map distances, in cM, are provided on the left side of the linkage groups. AFLP and SAMPL markers in bold and italics are intercross markers.

Five ESTP markers showed Mendelian segregation in the progeny. Three ESTP loci were heterozygous in the female parent and two were heterozygous in the male parent. These markers segregated in a 1:1 ratio. Two ESTP markers were mapped on LG XI as well as one each on three linkage groups I, V, and IX (Figures 1, 2, 3).

### Genetic linkage maps

The maternal map consisted of 816 markers distributed on 12 linkage groups covering 1,597 cM (Tables 5, 6). The number of mapped markers ranged from 35 to 93, with an average of 68 markers per linkage group. The length of the linkage groups ranged from 112 to 158 cM, with an average of 133 cM per linkage group (Table 6). The paternal map consisted of 743 markers assigned on 12 linkage groups, which covered 1,636 cM (Tables 5, 6). The number of mapped markers ranged from 25 to 89, with an average of 62 markers per linkage group. The length of the linkage groups ranged from 110 to 181 cM, with an average of 136 cM per linkage group (Table 6).

**Table 5:** Marker systems used for the construction of genetic linkage map and the number of markers mapped.

Marker type	Total number of markers mapped		
	
	Consensus map^1^	Maternal map	Paternal map
AFLP	809 (324)	577	556
SAMPL	255 (107)	201	161
SSR	42 (17)	35	24
ESTP	5 (0)	3	2

**Total**	**1,111 (448)**	**816**	**743**

^1^within-parentheses are the numbers of inter-cross or co-dominant markers used for construction of the consensus maps.

**Table 6:** Linkage groups, markers mapped, and marker density for the maternal, paternal, and consensus linkage maps in black spruce.

LG	Maternal map			Paternal map			Consensus map		
	
	Length (cM)	Markers		Length (cM)	Markers		Length (cM)	Markers	
						
		Total	Average/cM		Total	Average/cM		Total	Average/cM
I	123	93	1.3	125	81	1.5	132	122	1.1
II	126	79	1.6	139	73	1.9	149	108	1.4
III	121	72	1.7	181	83	2.2	131	104	1.3
IV	158	71	2.2	111	49	2.3	159	92	1.7
V	135	71	1.9	148	56	2.6	144	97	1.5
VI	137	81	1.7	116	78	1.5	144	105	1.4
VII	141	85	1.7	131	89	1.5	149	116	1.3
VIII	147	51	2.9	164	30	5.5	142	57	2.5
IX	135	71	1.9	129	62	2.1	172	100	1.7
X	127	35	3.6	127	42	3.0	156	66	2.4
XI	112	36	3.1	110	25	4.4	153	43	3.6
XII	135	71	1.9	155	75	2.1	139	101	1.4

**Total**	**1,597**	**816**	**2.0**	**1,636**	**743**	**2.2**	**1,770**	**1,111**	**1.6**

The homologous linkage groups between the parents were identified on the basis of segregating intercross AFLP, SAMPL, and SSR markers in the maternal and paternal maps. At least three intercross markers per linkage group were used. The integrated data set from the maternal and paternal maps allowed construction of a consensus linkage map. The consensus linkage map composed of 1,111 markers (Tables 5, 6) mapped to 12 linkage groups (Figures1, 2, 3). The linkage groups correspond to the haploid chromosome number (n = 12) of black spruce. It is worth noting that we have consistently obtained 12 linkage groups for the maternal, paternal, and consensus linkage maps, unlike other mapping studies where parental and/or consensus maps did not coalesce into 12 linkage groups. The consensus map covered 1,770 cM, with an average of 93 markers per linkage group and an average of one marker every 1.6 cM. The size of linkage groups varied from 131 to 172 cM, with an average of 148 cM (Table 6; Figures 1, 2, 3). The maternal, paternal and consensus genetic maps for each of the 12 linkage groups aligned very well and showed almost perfect colinearity of the marker order (Figure S1- Additional File 1).

### Genome length and map coverage

The estimated length of the black spruce genome was 1,786 cM based on the method of Chakravarti et al. [15], and 1,819 cM according to the method of Fishmann et al. [16]. The observed length of the black spruce genome obtained from the consensus genetic linkage map was 1,770 cM. Thus, the consensus genetic map constructed in our study covered more than 97% of the estimated genome length of black spruce.

### Distribution of markers along linkage groups

**Figure 4:**
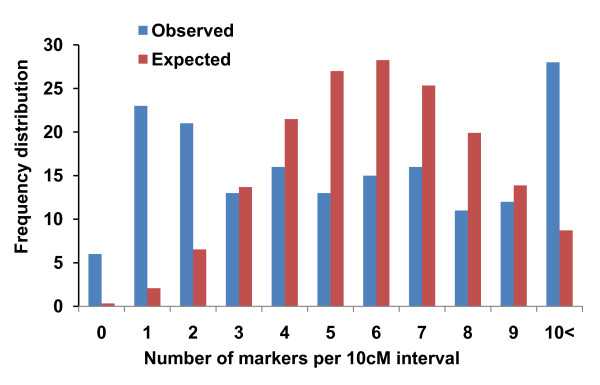
**Poisson distribution function for the observed and expected frequencies of the markers distributed at 10 cM interval**.

Significant deviations from the Poisson distribution of markers were observed for marker intervals of 2.5 cM, 5 cM, 10 cM, 20 cM, and 40 cM. For a 10 cM interval, the significant deviation (*P *< 0.001) is shown in Figure 4, indicating that the markers were not randomly distributed in the black spruce linkage groups. Marker distribution for other intervals (2.5 cM, 5 cM, 20 cM, and 40 cM) also showed clustering of markers (*P *< 0.05) along linkage groups. The independent analysis for testing the random distribution of AFLP (*P *< 0.001) and SAMPL (*P *< 0.001) markers indicated deviations from the random distribution. No correlation was observed between the number of mapped markers and the size of linkage groups. These results further support the clustering of makers on the linkage map.

**Figure 5:**
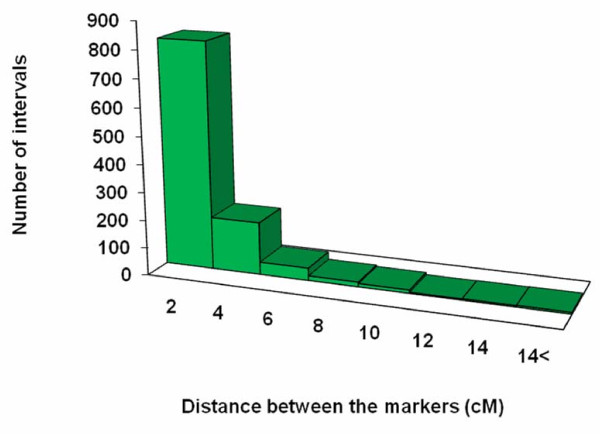
**Distribution of the map distance between two adjacent mapped markers**.

The distance between two adjacent markers on the linkage groups varied from 0 to 23.7 cM, with an average distance of 1.6 cM between any two adjacent markers (Figure 5; Table 6). This distance distribution reveals a strong skewness (*P *< 0.05), further indicating the non-random distribution of the markers along the linkage groups (Figure 5). Among the 1,099 intervals on 12 different linkage groups, 820 intervals were smaller than 2 cM (74.6%), and 89 intervals were larger than 6 cM (8.1%).

## Discussion

### Genetic linkage map

We have developed a high density to near-saturated and complete genetic linkage map of black spruce. This is the first genetic map for black spruce, although genetic maps have recently been reported for a black spruce × red spruce hybrid complex [12,13]. Except in the southern part of its range, red spruce is largely sympatric with black spruce. These two species hybridize in nature, although interspecific crossability represents a substantial but imperfect reproductive barrier for maintaining the separation of the species [17]. The differentiation of black and red spruce and their interspecific hybrids based on DNA markers, as used in Pelgas et al. [12] and Pavy et al. [13], is quite tenuous. The parents of the mapping pedigree in our study represent pure black spruce [18,19].

Our black spruce genetic map is the first map in the genus *Picea *based on a three-generation outbred pedigree (TGOP). There are only three other published genetic maps in the genus *Picea *that are based on pedigreed material: one each in Norway spruce and white spruce based on *F*_1 _mapping populations [9,11] and one in the black × red spruce hybrids based on *F*_1 _and *BC*_1 _mapping populations [12,13]. Almost all other reported maps are for single trees (Table 7). The single-tree genetic maps are based on segregation of markers in haploid megagametophytes of maternal trees and do not take into account the segregation or recombination of markers in the paternal trees. Also, in conifers, the recombination rates were reported to be lower in female gametes than those in male gametes [20,21]. Thus, single-tree linkage maps are not as informative as genetic maps developed from diploid segregating pedigreed populations, and are specific to a single tree (genotype), and thus, having limited or often no use for QTL mapping. Black spruce, like most other spruce or pine species, is highly outbred [22,23]. For genome and QTL mapping in outbred plants, TGOP is more informative than any other pedigree used so far in the genus *Picea *[14]. Indeed, TGOP not only allows differentiation of up to four segregating alleles at a locus but also establishment of linkage phase among alleles in the mapping population [14]. This information is required to use genetic linkage maps for QTL mapping. Furthermore, the outbred pedigrees are more representative of natural populations in an outbred plant. In *Pinus*, where the genome and QTL mapping work has been more advanced than in its sister genus *Picea*, high-density genetic and QTL maps have been prepared using TGOP in loblolly (*Pinus taeda *L.) and maritime (*Pinus pinaster *Aiton) pine [24-28].

**Table 7:** Comparison of the genetic linkage maps constructed in black spruce (*Picea mariana*) with those constructed (published) for other species in the genus *Picea*. Mega = megagametophytes.

Species	Mapping population	Mapping population size	Map type	No. markers mapped	Marker systems	No. linkage groups	Map length in cM (Kosambi)	Average distance between markers (cM)	Reference
***Picea mariana***	Three-generation outbred pedigree	90 *F*_2 _progeny	Maternal	816	AFLP,, SSR, SAMPL	12	1597	2.0	This study
			Paternal	743	ESTP	12	1636	2.2	
			Consensus	1,111		12	1770	1.6	

***Picea abies***	1 single tree	72 mega	Single tree	185	RAPD	17	3584	22.0	Binelli and Bucci 1994 [6]
	
	48 single trees	384 (48 × 8) mega	Population	70	RAPD	15			Bucci et al. 1997 [7]
	
	1 single tree	72 mega	Single tree	413	AFLP, SAMPL, SSR	29	2198.3	9.3	Paglia et al. 1998 [8]
	
	*F*_1_	73 *F*_1 _progeny	Maternal	461	AFLP, SSR,	12	1920	4.0	Acheré et al.
			Paternal	360	ESTP,	16	1792	4.9	2004 [9]
			Consensus	755	5srDNA	12	2035	2.6	

***Picea glauca***	1 single tree	47 mega	Single tree	47	RAPD	12	873.8	18.5	Tulsieram et al. 1992 [5]
	
	2 single trees	92 mega 96 mega	Single tree Single tree	165 144	RAPD, SCAR, ESTP	23 19	2059.4 2007.7		Gosselin et al. 2002 [10]
	
	*F*_1_	118 *F*_1 _progeny	Maternal	295	AFLP, SSR,	16 +3*	1842.3	6.2	Pelgas et al.
			Paternal	318	ESTP	15+4*	1928.2	6.1	2006 [11]
	*F*_1_	118 *F*_1 _progeny	Maternal	259		15+7*	1424.7	5.5	
			Paternal	264		12+8*	1533.6	5.8	
			Male consensus	512		13	1837.5	3.3	
	Composite *F*_1 _&*F*_1_	Composite *F*_1 _&*F*_1_	Composite	802		11	1933.5	2.4	

***Picea mariana *x *P. rubens *complex**	*F*_1_	80 *F*_1 _progeny	Maternal	326	AFLP, SSR,	15	1489.3	4.6	Pelgas et al.
			Paternal	303	ESTP, RAPD	20	1724.6	5.6	2005 [12]
	*BC*_1_	109 *BC*_1 _progeny	Maternal	313		14	1819.5	5.8	
			Paternal	281		17	1573.6	5.6	
			Male consensus	626		13	1704.8	2.8	
	Composite- *F*_1 _&*BC*_1_	Composite- *F*_1 _&*BC*_1_	Composite	1124		12	1845.5	1.6	
	
	*BC*_1_	283 *BC*_1 _progeny	Maternal	534	AFLP, ESTP, RAPD, SSR,	14	1833.5	4.0	Pavy et al. 2008 [13]
			Paternal	542	SNP	14	1814.1	3.8	
			Consensus	1,064		12	1849.8	2.2	

*minor linkage groups

### Genome length and map coverage

The black spruce genome length estimated in our study is 1,770 cM (Kosambi). This is comparable with the genome length of 1845.5 or 1849.8 cM (Kosambi) reported for black × red spruce hybrids based on a composite map of *F*_1 _and/or *BC*_1 _mapping populations (Table 7) [12,13] and that of 1,865 cM estimated for black × red spruce controlled cross hybrids from a *BC*_1 _mapping population (Kang et al. unpublished data). These results are consistent with the length of the genetic map for black spruce that is likely to be between 1800 and 1900 cM. The length of the black spruce genetic map observed in our study is about 15% shorter than that reported for Norway spruce (2,035 cM) [9] and white spruce (2,007, 2,059, or 1933.5 cM) [10,11] maps. The genome size (1C nuclear DNA contents) of Norway spruce (18.6 pg) [29] and white spruce (20.2 pg) [4] is 17.8% and 27.8%, respectively, higher than that of black spruce (15.8 pg) [4]. Although no direct relationships between the nuclear DNA contents and genetic map lengths apparently exist, the shorter genetic map length in black spruce than in Norway spruce or white spruce is apparently consistent with its comparatively smaller genome size. Our results suggest that the genome length observed in our study covers more than 97% of the estimated black spruce genome length. Thus, the black spruce genetic map reported here can very well be considered as almost complete. This is the highest map coverage so far for any *Picea *species. The estimated length and the extent of coverage of genetic maps in different species could vary owing to differences in the mapping populations used, variation in recombination rates of the parents, and the number and types of markers used in linkage map construction [14]. The mapping pedigree and markers used in our study are different from those used in Norway spruce and white spruce (Table 7).

### Linkage groups and marker density

In our study, the paternal, maternal, and consensus linkage maps consistently coalesced into 12 linkage groups, corresponding to the haploid chromosome number (n = 12) in black spruce. By contrast, in all other studies reported on genome mapping in the genus *Picea *(Table 7), with one exception, maternal, paternal, and/or consensus map did not coalesce into 12 linkage groups (see Table 7). The consensus map reported here has 1,111 markers distributed over 12 linkage groups, which represents an almost complete coverage of the black spruce genome, as the number of linkage groups corresponds to the haploid chromosome number. The average distance observed among adjacent markers mapped for the genetic map of black spruce in our study (1.6 cM) is comparable with or lower than that reported for the composite map of black spruce × red spruce complex [12,13], as well as lower than that reported for Norway spruce (2.6 cM) (Table 7). However, the marker density of the black spruce genetic map reported here is the highest for any genetic map based on a single cross in the genus *Picea*, as well for the maternal and paternal genetic maps (see Table 7 for comparisons).

### Marker systems

The genetic map of black spruce was constructed using AFLP, SAMPL, SSR, and ESTP marker systems. The AFLP and SAMPL systems provided a sufficient number of anonymous polymorphic and informative markers to construct a high density to near-saturated genetic map, whereas the SSR and ESTP systems provided highly informative and co-dominant markers. Although SSR and ESTP markers, due primarily to their codominant nature, would be preferred for genome mapping, availability of limited numbers of these markers precludes constructing a high density to saturated genetic map in conifers using only these markers. We mapped 809 AFLP markers that were resolved by 40 primer combination, showing an average multiplex ratio of 20 markers per primer combination. This multiplex ratio is comparable to that observed in Norway spruce (14) [9] and loblolly pine (21) [30]. We mapped 255 SAMPL markers on 12 different linkage groups. The multiplex ratio for SAMPL markers (21 polymorphic mapped markers per primer combinations) was comparable to that observed for AFLP markers. The only other report where SAMPL markers have been used for genetic linkage mapping in conifers is for Norway spruce [8], where 20 SAMPL markers were mapped using two primer combinations. A large number of AFLP and SAMPL markers segregated in the 3:1 ratio, which suggests that the parental genomes are highly heterozygous. As AFLP and SAMPL markers were dominant, the 3:1 segregating (intercross) markers were useful for aligning the parental maps to construct the consensus map, which cannot be established directly. Also the intercross markers can help to identify additional linkage groups that were not represented in the parental maps [31].

SSR loci provided highly informative markers. Forty-two SSR loci were mapped onto 11 linkage groups; 17 of these were highly informative for integrating the maternal and paternal maps to construct the consensus map. The mapped cDNA-based SSR markers are excellent candidates for comparative and composite mapping because these markers are expected to show high intraspecific homology and high interspecific orthology [e.g., [11]]. Also these markers allowed mapping of 10 known genes on seven different linkage groups.

Most EST primer pairs resolved multilocus patterns, which is not surprising given the occurrence of multigene families in conifers [32]. Also, the rate of polymorphism observed in ESTPs was very low, which suggests that more powerful methods, such as detection of single nucleotide polymorphisms (SNPs) can be used to increase the resolution of polymorphism [13]. Nevertheless, the mapped ESTP loci along with cDNA-based SSR markers provide good candidates for comparative mapping in *Picea*, Pinaceae, or other conifers [12,33,34].

The genetic map presented here is the first-generation genetic map for black spruce, which provides a framework to map SNP and other markers in the second-generation genetic map. We are planning to map SNP markers from candidate genes and genes expressed differentially in response to climate change and SNPs mapped by Pavy et al. [13] onto this map in future. This is further discussed below under the Future Perspective section. The mapped SNP markers from expressed genes are quite useful for QTL and association mapping of relevant traits because allelic variation in the genes could be linked or associated with trait phenotypes. However, these markers represent a very small proportion of the spruce genome. Assuming 50,000 expressed genes of average 1 kb size and genome size of 30 Gbp in black spruce, the transcribed genome represents less than 0.2% of the whole genome. SNPs in about 500 candidate genes are normally mapped, further reducing the proportion of the genome sampled to less than 0.002%, although the mapped genes may have genome-wide distribution. The anonymous markers, such as AFLP and SAMPL markers generally provide a whole-genome scan; thus a genetic map using these markers may cover a large proportion of the genome, if not the whole genome. However, anonymous markers such as AFLP and SAMPL, have limitations in tagging genes controlling traits of interest via QTL and association mapping because variation in these markers may not represent functional genetic variation. Nevertheless, the black spruce genetic map reported here provides a valuable genomic resource in *Picea *and Pinaceae.

### Clustering of markers

Even though only those markers segregating in Mendelian ratios and not those showing a distorted segregation were used for the linkage analysis, clustering of AFLP and SAMPL markers was detected in the linkage groups of black spruce. These results agree with the clustering of AFLP markers reported for genetic maps of *Picea abies *[35], *Pinus taeda *[30], and *Pinus sylvestris*L[36], but in contrast to random distribution of AFLP markers in the genetic maps reported for Norway spruce [9] and black × red spruce hybrids [12]. It should be noted that the study of Scotti et al. [35] was specifically performed to examine the distribution of marker classes in a genetic linkage map of Norway spruce.

The non-random distribution of markers may be caused by non-random and unequal crossing over and recombination along the chromosome length. The recombination is suppressed in the centromeric and heterochromatic pericentromeric regions [37], and the presence of heterochromatin in pericentromeric regions is a general feature of plant chromosomes. Assuming a random distribution of markers, low levels of meiotic recombination may well cause markers that are physically well separated, to cluster on a linkage map.

## Future Perspectives

The genetic map reported here is suitable for constructing a composite map of two TGOP in black spruce and for the envisaged comparative mapping with red spruce and black spruce × red spruce hybrid. The mapped EST-based microsatellites and ESTPs will provide very useful markers for this work. We are also mapping additional EST-based SSR markers. Black spruce and red spruce are highly genetically related species but their evolutionary relationships are controversial. Comparative genome mapping may shed some light on comparative genome organization (orthology, synteny and order of the markers) and evolution of these species. The map could also be used for comparative mapping with other genera of Pinaceae.

We are genotyping and phenotyping the larger mapping populations of the TGOP used in this study and another TGOP (> 300) of black spruce for QTL mapping of traits related to growth and adaptation to climate change. We plan to use about 1,500 SNPs from candidate genes and genes expressed differentially in response to climate change conditions as well as SNPs mapped by Pavy et al. [13] for our QTL mapping work, using a high throughput SNP genotyping platform such as Illumina's Golden Gate Genotyping Assay http://www.illumina.com/technology/goldengate_genotyping_assay.ilmn. Thus, the technologies, markers and genetic map developed in the present study provide an invaluable genomic resource for basic and applied genomics studies.

## Conclusions

We have constructed the first high density to near-saturated genetic linkage map of black spruce, with above 97% genome coverage. The maternal, paternal, and consensus maps in our study consistently coalesced into 12 linkage groups, corresponding to the haploid chromosome number (1n = 1x = 12) of 12 in the genus *Picea*. Also, this is the first genetic map based on a three-generation outbred pedigree in the genus *Picea*. The genome length in *P. mariana *is likely to be about 1,800 cM. The genetic maps developed in our study can serve as a reference map for various genomics studies and applications in *Picea a*nd Pinaceae. It will provide a foundation and a valuable resource for comparative mapping, constructing composite maps, and mapping quantitative trait loci and determining the genetic basis of complex quantitative traits of interest, such as growth and adaptation to climate change.

## Methods

### Mapping population

A three generation outbred pedigree (TGOP), including the grandparents, parents (*F*_1_), and *F*_2 _progeny, was used to construct the black spruce genetic linkage map. The grandparents of this pedigree were part of a 7 × 7 diallel *F*_1 _controlled-cross experiment, performed by Dr. E.K. Morgenstern in the early 1970s at the Petawawa National Forestry Institute (PNFI), in Chalk River, Ontario, Canada (46° N, 77° 30' W) [18]. The seven parental trees used for the diallel cross were from a pure black spruce plantation established at the Petawawa Research Forest (PRF), but the exact origin of the trees is unknown, other than that they were grown from seeds collected from the Lake Simcoe-Rideau region in Ontario [18]. The *F*_1 _seedlings from the full-sib families of this diallel were planted in genetic tests at three sites at PRF in 1973 [18]. The parents of the mapping pedigree were crossed from the *F*_1 _genetic tests in 1987 and 1988 by Dr. Tim Boyle at PNFI to produce *F*_2 _controlled crosses [19]. The *F*_2 _family 643 (32 × 40) was selected for genetic mapping purposes based on near-top and bottom ranking of its parents for growth and ^13^C discrimination rate [38] and availability of sufficient number of *F*_2 _seeds. Grandparents, parents, and 90 *F*_2 _individuals from this family were used as the mapping population. The *F*_2 _progeny were raised and grown at the Canadian Forest Service-Atlantic Forestry Centre, Fredericton, New Brunswick, Canada (45° 52' N, 66° 31' W).

### DNA extraction

Genomic DNA was extracted from needle tissues of the female grandparent and megagametophtyes of the male grandparent, and needle tissues of the parents and their *F*_2 _progenies, using the Qiagen DNeasy Plant^® ^Mini Kit, following the manufacturer's protocol (Qiagen Inc. Mississauga, ON, Canada). Needle tissues from the paternal grandparent were not available as the tree was harvested from the plantation, but its open-pollinated seeds were stored at the Atlantic Forestry Centre. Therefore, to genotype this grandparent, we used DNA extracted from pooled megagametophyte tissues from 20 to 30 seeds. In spruce, the genetic constitution of haploid megagametophytes is the same as that of female gametes of the mother tree. The quality and quantity of DNA preparations were determined by electrophoresing the DNA samples along with a standard of Lambda DNA on 0.8% agarose gels followed by staining with ethidium bromide.

### Marker systems

Four different marker systems were used to genotype the grandparents, parents, and *F*_2 _progeny of the TGOP: AFLPs, SAMPL, microsatellites or SSRs, and ESTPs. We used four marker systems in order to achieve better genome coverage because different marker types target different genomic regions.

### AFLP markers

Because of the extremely large genome size of spruce, a standard AFLP protocol, based on *Eco*RI-*Mse*I digestion, *Eco*RI, and *Mse*I primer extension by 1 nucleotide in the preamplification step and 3 base extension to the *Eco*RI and *Mse*I primers in the selective amplification step [39], produced complex AFLP fragment patterns. We developed methods for high throughput resolution of high-quality and clearly scorable AFLP markers for black spruce, using LI-COR 4200L^® ^(LI-COR Biosciences, Lincoln, NE, USA) or Beckmann Coulter CEQ 8000 GENETIC ANALYSIS SYSTEM^® ^(Beckmann Coulter, Fullerton, CA, USA), by evaluating a variety of conditions, including *Eco*RI and *Mse*I restriction digestion time of black spruce genomic DNA, and the number of selective nucleotides used in the preamplification and selective amplification steps. The primer combinations producing consistent, clear, and easily scorable polymorphic AFLP markers were identified and used for genotyping the mapping population.

The AFLP method was essentially as described in Vos et al. [39], with some modifications. Black spruce genomic DNA (500 ng) was digested with 2U each of *Eco*RI and *Mse*I (New England Biolab Inc. Ipswich, MA, USA) for 3 h at 37°C, followed by incubation at 70°C for 20 min. The digested DNA was ligated overnight with the *Eco*RI and *Mse*I adapters in a total volume of 20 μl at 25°C, followed by incubation at 70°C for 20 min. This restriction-ligation (RL) mixture was diluted 1:5 with autoclaved deionized distilled water before using it in the preamplification step.

A 3 μl aliquot of the RL mixture was preamplified using *Eco*RI (E) and *Mse*I (M) preamplification primers with an extension of one or two selective nucleotides at the 3' end. *Eco*RI preamp primers (+1/+2):

(+1) 5'**- **GAC TGC GTA CCA ATT C**A **- 3'

(+2) 5'**- **GAC TGC GTA CCA ATT C**AC **-3'

*Mse*I preamp primers (+1/+2):

(+1) 5'-GAT GAG TCC TGA GTA **AC **- 3'

(+2) 5'-GAT GAG TCC TGA GTA A**CC **- 3'

The PCR profile for the preamplification step consisted of 20 cycles each of denaturation at 94°C for 30 sec, annealing at 56°C for 1 min, and extension at 72°C for 1 min, followed by a final soak at 10°C using a PTC-200 thermal cycler (MJ Research, Reno, NV, USA). After the preamplification step, the reaction mixture was diluted 1:50 with sterile deionized distilled water. A total of 54 different *Eco*RI and *Mse*I primer pairs were tested with one or two selective nucleotides at the preamplification step and three to five selective nucleotides at the selective amplification step. From these, 40 AFLP primer combinations were selected for further use in mapping. Selective amplifications were performed using these primer combinations with various selective nucleotide extensions (E+3/M+3, E+3/M+4, E+3/M+5) (Table 1). The reaction mixture for the selective amplification consisted of 2 μl of diluted preamplified template DNA, 1 U *Taq *polymerase, 2.5 ng of *Eco*RI labeled primer (IRDye 700 label for LI-COR and D2 or D3 label for Beckmann Coulter CEQ 8000 GENETIC ANALYSIS SYSTEM), 12.5 ng *Mse*I primer, 10× PCR buffer (MBI Fermentas Inc, Burlington, ON, Canada), 1.5 mM MgCl_2_, 0.2 mM each of all four dNTPs (MBI Fermentas Inc, Burlington, ON, Canada), and BSA (1 μg/μl) (Sigma-Aldrich, Oakville, ON, Canada). PCR amplification profile consisted of 12 cycles each of denaturation at 94°C for 30 sec, annealing at 65°C for 30 sec (with lowering of 0.7°C per cycle) and extension at 72°C for 1 min, followed by 23 cycles each of denaturation at 94°C for 30 sec, annealing at 56°C for 60 sec and extension at 72°C for 1 min, followed by a final soak at 10°C.

Reaction products following selective amplification were resolved either on LI-COR 4200L or Beckmann Coulter CEQ 8000 GENETIC ANALYSIS SYSTEM. For LI-COR, selective amplification products were resolved on 6.5% denaturing Long Ranger polyacrylamide gels (LI-COR Biosciences, Lincoln, NE, USA). Approximately 0.5 μl of each sample (10 μl of PCR product and 15 ul of loading dye) was loaded on the gel. IRD-labeled molecular-weight markers were loaded in three lanes as a size-standard. Electrophoresis was carried out using 1× TBE running buffer, with run parameters of 1500 V, 35 mA, 70 W, signal channel 3, motor speed 3, 50°C plate temperature and 16-bit pixel depth for collection of TIFF image files. Polymorphic fragments were visually scored in the TIFF image files. Only those markers that were segregating in a Mendelian ratio (*χ^2^*- test, *P *< 0.05) were scored. For Beckmann Coulter CEQ 8000 GENETIC ANALYSIS SYSTEM, 2 μl of the selective amplification product was added to 27.5 μl of sample loading solution and 0.5 μl of CEQ DNA size standard-600 (Beckmann Coulter, Fullerton, CA, USA), followed by overlaying a drop of mineral oil. Samples were injected into a 33 cm capillary at 2.0 KV for 90 sec and electrophoresed at 7.5 KV for 70 min at 35°C. The AFLP fragments data were exported to an Excel^® ^file using fragment analysis software for further analysis of genetic linkage parameters.

### SAMPL markers

Selectively amplified microsatellite polymorphic loci markers, based on a combination of AFLP and microsatellite technology, can combine good features of both AFLP and microsatellite markers, and can reduce the marker complexity of AFLPs in spruce. The SAMPL technology is a modified AFLP technique, in which a compound microsatellite sequence is used as one of the two AFLP primers in selective amplification, generally in place of *Eco*RI primers [40]. We developed SAMPL markers using the compound microsatellite repeats from *Lactuca *species [41] as SAMPL primers (Table 2) in place of the *Eco*RI primer in the selective amplification step. The SAMPL markers were analyzed on the LI-COR and Beckman CEQ 8000 systems, using the protocol described above for AFLP analysis as well as in Gupta et al. [40].

Sixteen combinations of four SAMPL and four *Mse*I primers (with an extension of three selective nucleotides) were tested to screen SAMPL marker polymorphisms between the parents of the mapping population. Of these, 12 primer combinations were selected for genotyping of the mapping population based on the quality and polymorphism of the markers resolved (Table 3). The SAMPL marker data were scored as described above for AFLP markers.

### Microsatellite/SSR markers

Seventy-eight microsatellites developed from black spruce cDNA (EST) or genomic DNA sequences and white spruce ESTs in our lab were used to screen polymorphisms between the parents of the mapping population. Forty-two microsatellites showed inter-parental polymorphisms, and were used to genotype the mapping population (Table 4). Out of these, 20 were from the white spruce ESTs, 6 from black spruce ESTs, and 16 from black spruce genomic sequences (SSR-enriched and AFLP-SSR libraries). White spruce EST sequences were obtained from the publicly available NCBI GenBank EST database. EST sequences containing microsatellites were identified. Primers were designed and microsatellite markers were optimized. The mapped six black spruce EST-SSRs were developed from the EST sequences obtained from a cambium-transcript enriched cDNA library constructed from the male parent (40) of the mapping population. The details of the study on cDNA library construction and EST sequencing, analysis and annotation will be published elsewhere. Here, we provide the primer sequences and annealing temperatures for six mapped EST-SSR markers in the Additional File 2. The results on the development and characterization of microsatellite DNA markers from the white spruce EST sequences (RPGSE series) and from the black spruce genomic sequences (RPMSA and RPMSG series) will be published elsewhere because this work was performed by other researchers in the Rajora lab. However, pending publication, information on these mapped markers, including primer sequences, is available from the Principal Investigator O.P. Rajora of the Spruce Genomics Program. The microsatellite markers were resolved on the LI-COR system and data scored as described in Rajora et al. [42,43].

### ESTP markers

Primers for 198 ESTs, obtained from sequencing of a black spruce standard cDNA library prepared from needle tissue (to be published separately), were designed using Primer 3.0 software [44]. The parents of the mapping population were screened for ESTPs (length polymorphism). Most EST primers resolved multilocus patterns. Only five ESTP markers, showing inter-parental polymorphism, displayed unambiguous single-locus patterns and therefore, were used to genotype the mapping population. The primer sequences and annealing temperatures for these mapped ESTP markers are provided in the Additional File 2. The PCR amplification profile consisted of initial denaturation at 94°C for 5 min, 40 cycles each of denaturation at 94°C for 1 min, annealing at 55°C for 1 min, and extension at 72°C for 1.3 min, followed by final extension at 72°C for 10 min. The ESTP markers were resolved by electrophoresis on either 2% agarose or 6% polyacrylamide gels.

### Nomenclature or labeling of markers on the genetic maps

The AFLP and SAMPL markers were named, starting with letters A, and S, respectively, followed by the primer number, and then the size of the fragment. The AFLP and SAMPL fragment sizes reported in this manuscript are without adapter sequences. The SSR markers were named with a prefix of five letters. The first letter represents the Principal Investigator/Lab (R = Rajora), the next two letters the species name (PG = *Picea glauca*, PM = *Picea mariana*) from which the markers were developed, the next letter S representing SSR, and the last letter denoting the source of sequences or library type (E = EST; G = Genomic; A = AFLP-SSR genomic). These prefix letters were followed by the marker number. Thus, SSR markers developed from the white spruce EST database have a prefix of RPGSE, SSR markers developed from black spruce EST sequences a prefix of RPMSE, SSR markers developed from the genomic library a prefix of RPMSG, and SSR markers developed from the black spruce SSR-enriched AFLP sequences a prefix of RPMSA. The ESTP markers developed from the EST sequences from the black spruce cDNA library were named starting with RPMEP, followed by the marker number.

### Statistical analysis

#### Segregation analysis and map construction

Individual paternal and maternal maps were constructed according to two-way pseudo-testcross mapping strategy [45]. All linkage analysis and genetic map construction, including marker order and map length estimations, were performed using JOINMAP^® ^3.0 software [46] with the maximal threshold jump value of 5 and ripple value of 1. The Kosambi [47] mapping function was used for map length estimations. Our marker data set for genetic linkage mapping included three different segregation patterns: 1:1 for markers heterozygous in one parent and homozygous or null in the other, 3:1 for dominant markers heterozygous in both parents, and 1:2:1 or 1:1:1:1 for co-dominant markers heterozygous in both parents. The JOINMAP command "similarity of loci" was used to identify the similar loci. Only one of the markers was kept from the similar loci for linkage mapping analysis.

For framework, preliminary linkage grouping of AFLPs, SAMPL, SSRs, and ESTPs markers were ordered using the "Group" command, with LOD (log of odds) threshold maximum 5.0, minimum 4.0, recombination ratio 0.35. The map orders were found by calculating pairwise recombination frequencies, and map distances were estimated by a least-squares procedure. The two parental maps based on segregating markers were grouped and ordered using a minimum LOD score of 3.0 and recombination fraction of 0.4 as the grouping criterion. The marker order obtained from the third round of analysis was retained with the JOINMAP command "calculate map". This order was fixed to allow positioning of additional markers. To construct consensus maps, the maternal maps and paternal were aligned based on the co-dominant markers (1:2:1, 1:1:1:1 and 1:1) and intercross markers (3:1), and then consensus maps were constructed by using JoinMap function "Combine Groups for Map Integration" command. The maternal, paternal and consensus genetic maps were aligned using the JOINMAP. Graphic presentation of the individual linkage groups was drawn using Mapchart^® ^version 2.0 software [48].

#### Estimation of genome length and map coverage

The length of the black spruce genome (*G*) was estimated using the Method 4 of Chakravarti et al. [15] after each length had been adjusted by the factor *m+1/m-1*, where m is the number of framework markers on the linkage group, as well by the method described by Fishmann et al. [16] that twice the value of map density (*d*) was added to the length of each linkage group to account for chromosome ends beyond the terminal markers, and *G *was calculated by summing up the resulting lengths of 12 linkage groups. The observed genome length was obtained by summing up the map lengths of the 12 individual linkage groups. The map coverage was calculated as the ratio of the observed to the estimated genome length. The number of markers required to cover the whole genome of black spruce was calculated according to Lange and Boehnke [49].

#### Marker distribution analysis

To evaluate whether the mapped markers were randomly distributed on the linkage map, the linkage groups were divided into 2.5, 5, 10, 20, and 40 cM blocks, and the number of markers per block was counted. Observed frequencies of the number of markers per block were compared with the expected ones by performing a Chi-square test [30,36,50], using a Poisson distribution function, *P(x) *= e*^-μ^*μ*^x^*/*x*!, where *x *is the number of markers per block and μ is the average marker density in the consensus map. Average marker density (μ) was used to calculate the expected binomial frequencies for each marker class per block interval for all the linkage groups. The distribution of markers on the linkage groups was also evaluated separately for the AFLP and SAMPL markers. The SSR and ESTP markers could not be considered independently for this analysis because of their small numbers or low frequencies on each linkage group.

## Authors' contributions

BYK: SAMPL genotyping, data analysis and construction and alignment of the genetic maps; IKM: AFLP and ESTP genotyping, early data analysis and genetic map construction, preparation of preliminary drafts of the parts of the manuscript; JEM: mapping population and its selection, propagation and sampling, and manuscript revision; OPR: project conception, design, funding, guidance and overall supervision, manuscript preparation and revision. All authors have read and approved the manuscript.

## Supplementary Material

Additional file 1 **Figure S1 Alignment of the maternal (Maternal), paternal (Paternal) and consensus (PM643) linkage maps of black spruce**. Names of the markers are provided on the right side of the linkage groups, with the DNA fragment size in bp. Genetic map distances, in cM, are provided on the left side of the linkage groups. AFLP markers start with A, SAMPL markers with S. The SSR and ESTP markers are italicized and are in red color. Click here for file

Additional file 2 **Table S1 Expressed sequence tag polymorphism (ESTP) and EST-based microsatellite (SSR) markers developed from black spruce ESTs and mapped on the black spruce genetic map in this study**. Click here for file
