# Supplementary material for: Near-saturated and complete genetic linkage map of black spruce (Picea mariana)
Source: BMC Genomics. 2010 Sep 24;11:515. doi: 10.1186/1471-2164-11-515 (PMC2997009; doi:10.1186/1471-2164-11-515)
Supplement: Additional file 1 — Figure S1 Alignment of the maternal (Maternal), paternal (Paternal) and consensus (PM643) linkage maps of black spruce. Names of the markers are provided on the right side of the linkage groups, with the DNA fragment size in bp. Genetic map distances, in cM, are provided on the left side of the linkage groups. AFLP markers start with A, SAMPL markers with S. The SSR and ESTP markers are italicized and are in red color. [file 1471-2164-11-515-S1.PDF]

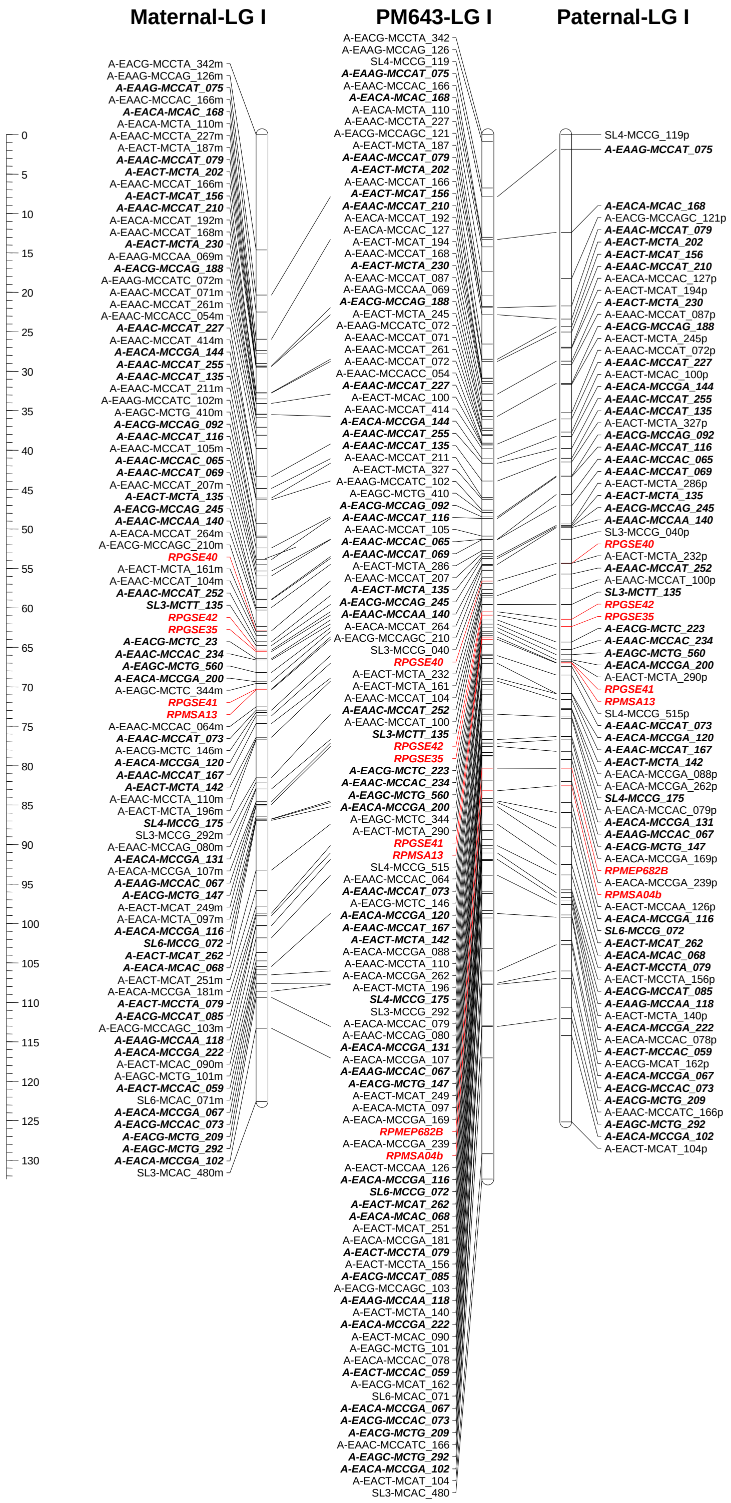
Maternal-LG I
PM643-LG I
Paternal-LG I
0
5
10
15
20
25
30
35
40
45
50
55
60
65
70
75
80
85
90
95
100
105
110
115
120
125
130
A-EACG-MCCTA_342m
A-EAAG-MCCAG_126m
A-EAAG-MCCAT_075
A-EAAC-MCCAC_166m
A-EACA-MCAC_168
A-EACA-MCTA_110m
A-EAAC-MCCTA_227m
A-EACT-MCTA_187m
A-EAAC-MCCAT_079
A-EACT-MCTA_202
A-EAAC-MCCAT_166m
A-EACT-MCAT_156
A-EAAC-MCCAT_210
A-EACA-MCCAT_192m
A-EAAC-MCCAT_168m
A-EACT-MCTA_230
A-EAAG-MCCAA_069m
A-EACG-MCCAG_188
A-EAAG-MCCATC_072m
A-EAAC-MCCAT_071m
A-EAAC-MCCAT_261m
A-EAAC-MCCACC_054m
A-EAAC-MCCAT_227
A-EAAC-MCCAT_414m
A-EACA-MCCGA_144
A-EAAC-MCCAT_255
A-EAAC-MCCAT_135
A-EAAC-MCCAT_211m
A-EAAG-MCCATC_102m
A-EAGC-MCTG_410m
A-EACG-MCCAG_092
A-EAAC-MCCAT_116
A-EAAC-MCCAT_105m
A-EAAC-MCCAC_065
A-EAAC-MCCAT_069
A-EAAC-MCCAT_207m
A-EACT-MCTA_135
A-EACG-MCCAG_245
A-EAAC-MCCAA_140
A-EACA-MCCAT_264m
A-EACG-MCCAGC_210m
RPGSE40
A-EACT-MCTA_161m
A-EAAC-MCCAT_104m
A-EAAC-MCCAT_252
SL3-MCTT_135
RPGSE42
RPGSE35
A-EACG-MCTC_23
A-EAAC-MCCAC_234
A-EAGC-MCTG_560
A-EACA-MCCGA_200
A-EAGC-MCTC_344m
RPGSE41
RPMSA13
A-EAAC-MCCAC_064m
A-EAAC-MCCAT_073
A-EACG-MCTC_146m
A-EACA-MCCGA_120
A-EAAC-MCCAT_167
A-EACT-MCTA_142
A-EAAC-MCCTA_110m
A-EACT-MCTA_196m
SL4-MCCG_175
SL3-MCCG_292m
A-EAAC-MCCAG_080m
A-EACA-MCCGA_131
A-EACA-MCCGA_107m
A-EAAG-MCCAC_067
A-EACG-MCTG_147
A-EACT-MCAT_249m
A-EACA-MCTA_097m
A-EACA-MCCGA_116
SL6-MCCG_072
A-EACT-MCAT_262
A-EACA-MCAC_068
A-EACT-MCAT_251m
A-EACA-MCCGA_181m
A-EACT-MCCTA_079
A-EACG-MCCAT_085
A-EACG-MCCAGC_103m
A-EAAG-MCCAA_118
A-EACA-MCCGA_222
A-EACT-MCAC_090m
A-EAGC-MCTG_101m
A-EACT-MCCAC_059
SL6-MCAC_071m
A-EACA-MCCGA_067
A-EACG-MCCAC_073
A-EACG-MCTG_209
A-EAGC-MCTG_292
A-EACA-MCCGA_102
SL3-MCAC_480m
A-EACG-MCCTA_342
A-EAAG-MCCAG_126
SL4-MCCG_119
A-EAAG-MCCAT_075
A-EAAC-MCCAC_166
A-EACA-MCAC_168
A-EACA-MCTA_110
A-EAAC-MCCTA_227
A-EACG-MCCAGC_121
A-EACT-MCTA_187
A-EAAC-MCCAT_079
A-EACT-MCTA_202
A-EAAC-MCCAT_166
A-EACT-MCAT_156
A-EAAC-MCCAT_210
A-EACA-MCCAT_192
A-EACA-MCCAC_127
A-EACT-MCAT_194
A-EAAC-MCCAT_168
A-EACT-MCTA_230
A-EAAC-MCCAT_087
A-EAAG-MCCAA_069
A-EACG-MCCAG_188
A-EACT-MCTA_245
A-EAAG-MCCATC_072
A-EAAC-MCCAT_071
A-EAAC-MCCAT_261
A-EAAC-MCCAT_072
A-EAAC-MCCACC_054
A-EAAC-MCCAT_227
A-EACT-MCAC_100
A-EAAC-MCCAT_414
A-EACA-MCCGA_144
A-EAAC-MCCAT_255
A-EAAC-MCCAT_135
A-EAAC-MCCAT_211
A-EACT-MCTA_327
A-EAAG-MCCATC_102
A-EAGC-MCTG_410
A-EACG-MCCAG_092
A-EAAC-MCCAT_116
A-EAAC-MCCAT_105
A-EAAC-MCCAC_065
A-EAAC-MCCAT_069
A-EACT-MCTA_286
A-EAAC-MCCAT_207
A-EACT-MCTA_135
A-EACG-MCCAG_245
A-EAAC-MCCAA_140
A-EACA-MCCAT_264
A-EACG-MCCAGC_210
SL3-MCCG_040
RPGSE40
A-EACT-MCTA_232
A-EACT-MCTA_161
A-EAAC-MCCAT_104
A-EAAC-MCCAT_252
A-EAAC-MCCAT_100
SL3-MCTT_135
RPGSE42
RPGSE35
A-EACG-MCTC_223
A-EAAC-MCCAC_234
A-EAGC-MCTG_560
A-EACA-MCCGA_200
A-EAGC-MCTC_344
A-EACT-MCTA_290
RPGSE41
RPMSA13
SL4-MCCG_515
A-EAAC-MCCAC_064
A-EAAC-MCCAT_073
A-EACG-MCTC_146
A-EACA-MCCGA_120
A-EAAC-MCCAT_167
A-EACT-MCTA_142
A-EACA-MCCGA_088
A-EAAC-MCCTA_110
A-EACA-MCCGA_262
A-EACT-MCTA_196
SL4-MCCG_175
SL3-MCCG_292
A-EACA-MCCAC_079
A-EAAC-MCCAG_080
A-EACA-MCCGA_131
A-EACA-MCCGA_107
A-EAAG-MCCAC_067
A-EACG-MCTG_147
A-EACT-MCAT_249
A-EACA-MCTA_097
A-EACA-MCCGA_169
RPMEP682B
A-EACA-MCCGA_239
RPMSA04b
A-EACT-MCCAA_126
A-EACA-MCCGA_116
SL6-MCCG_072
A-EACT-MCAT_262
A-EACA-MCAC_068
A-EACT-MCAT_251
A-EACA-MCCGA_181
A-EACT-MCCTA_079
A-EACT-MCCTA_156
A-EACG-MCCAT_085
A-EACG-MCCAGC_103
A-EAAG-MCCAA_118
A-EACT-MCTA_140
A-EACA-MCCGA_222
A-EACT-MCAC_090
A-EAGC-MCTG_101
A-EACA-MCCAC_078
A-EACT-MCCAC_059
A-EACG-MCAT_162
SL6-MCAC_071
A-EACA-MCCGA_067
A-EACG-MCCAC_073
A-EACG-MCTG_209
A-EAAC-MCCATC_166
A-EAGC-MCTG_292
A-EACA-MCCGA_102
A-EACT-MCAT_104
SL3-MCAC_480
SL4-MCCG_119p
A-EAAG-MCCAT_075
A-EACA-MCAC_168
A-EACG-MCCAGC_121p
A-EAAC-MCCAT_079
A-EACT-MCTA_202
A-EACT-MCAT_156
A-EAAC-MCCAT_210
A-EACA-MCCAC_127p
A-EACT-MCAT_194p
A-EACT-MCTA_230
A-EAAC-MCCAT_087p
A-EACG-MCCAG_188
A-EACT-MCTA_245p
A-EAAC-MCCAT_072p
A-EAAC-MCCAT_227
A-EACT-MCAC_100p
A-EACA-MCCGA_144
A-EAAC-MCCAT_255
A-EAAC-MCCAT_135
A-EACT-MCTA_327p
A-EACG-MCCAG_092
A-EAAC-MCCAT_116
A-EAAC-MCCAC_065
A-EAAC-MCCAT_069
A-EACT-MCTA_286p
A-EACT-MCTA_135
A-EACG-MCCAG_245
A-EAAC-MCCAA_140
SL3-MCCG_040p
RPGSE40
A-EACT-MCTA_232p
A-EAAC-MCCAT_252
A-EAAC-MCCAT_100p
SL3-MCTT_135
RPGSE42
RPGSE35
A-EACG-MCTC_223
A-EAAC-MCCAC_234
A-EAGC-MCTG_560
A-EACA-MCCGA_200
A-EACT-MCTA_290p
RPGSE41
RPMSA13
SL4-MCCG_515p
A-EAAC-MCCAT_073
A-EACA-MCCGA_120
A-EAAC-MCCAT_167
A-EACT-MCTA_142
A-EACA-MCCGA_088p
A-EACA-MCCGA_262p
SL4-MCCG_175
A-EACA-MCCAC_079p
A-EACA-MCCGA_131
A-EAAG-MCCAC_067
A-EACG-MCTG_147
A-EACA-MCCGA_169p
RPMEP682B
A-EACA-MCCGA_239p
RPMSA04b
A-EACT-MCCAA_126p
A-EACA-MCCGA_116
SL6-MCCG_072
A-EACT-MCAT_262
A-EACA-MCAC_068
A-EACT-MCCTA_079
A-EACT-MCCTA_156p
A-EACG-MCCAT_085
A-EAAG-MCCAA_118
A-EACT-MCTA_140p
A-EACA-MCCGA_222
A-EACA-MCCAC_078p
A-EACT-MCCAC_059
A-EACG-MCAT_162p
A-EACA-MCCGA_067
A-EACG-MCCAC_073
A-EACG-MCTG_209
A-EAAC-MCCATC_166p
A-EAGC-MCTG_292
A-EACA-MCCGA_102
A-EACT-MCAT_104p

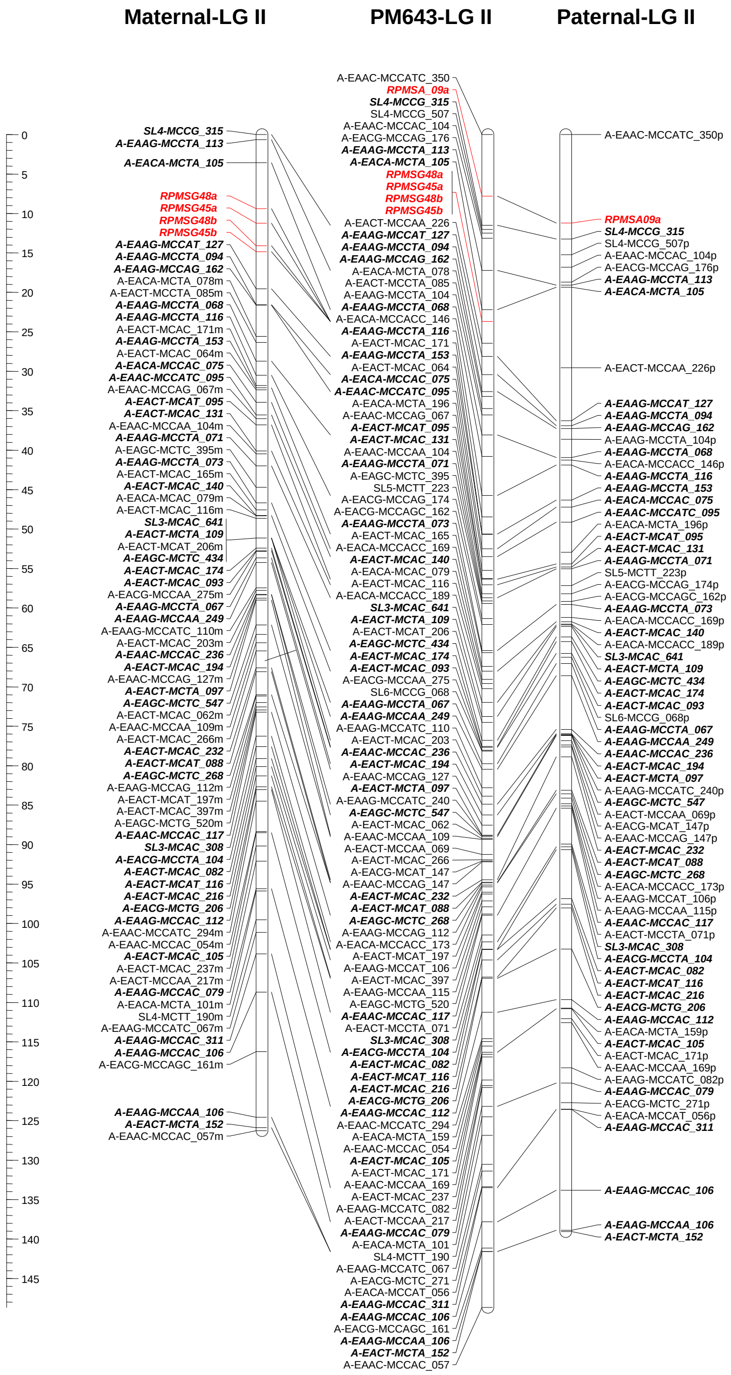
Maternal-LG II
PM643-LG II
Paternal-LG II
0
5
10
15
20
25
30
35
40
45
50
55
60
65
70
75
80
85
90
95
100
105
110
115
120
125
130
135
140
145
SL4-MCCG_315
A-EAAG-MCCTA_113
A-EACA-MCTA_105
RPMSG48a
RPMSG45a
RPMSG48b
RPMSG45b
A-EAAG-MCCAT_127
A-EAAG-MCCTA_094
A-EAAG-MCCAG_162
A-EACA-MCTA_078m
A-EACT-MCCTA_085m
A-EAAG-MCCTA_068
A-EAAG-MCCTA_116
A-EACT-MCAC_171m
A-EAAG-MCCTA_153
A-EACT-MCAC_064m
A-EACA-MCCAC_075
A-EAAC-MCCATC_095
A-EAAC-MCCAG_067m
A-EACT-MCAT_095
A-EACT-MCAC_131
A-EAAC-MCCAA_104m
A-EAAG-MCCTA_071
A-EAGC-MCTC_395m
A-EAAG-MCCTA_073
A-EACT-MCAC_165m
A-EACT-MCAC_140
A-EACA-MCAC_079m
A-EACT-MCAC_116m
SL3-MCAC_641
A-EACT-MCTA_109
A-EACT-MCAT_206m
A-EAGC-MCTC_434
A-EACT-MCAC_174
A-EACT-MCAC_093
A-EACG-MCCAA_275m
A-EAAG-MCCTA_067
A-EAAG-MCCAA_249
A-EAAG-MCCATC_110m
A-EACT-MCAC_203m
A-EAAC-MCCAC_236
A-EACT-MCAC_194
A-EAAC-MCCAG_127m
A-EACT-MCTA_097
A-EAGC-MCTC_547
A-EACT-MCAC_062m
A-EAAC-MCCAA_109m
A-EACT-MCAC_266m
A-EACT-MCAC_232
A-EACT-MCAT_088
A-EAGC-MCTC_268
A-EAAG-MCCAG_112m
A-EACT-MCAT_197m
A-EACT-MCAC_397m
A-EAGC-MCTG_520m
A-EAAC-MCCAC_117
SL3-MCAC_308
A-EACG-MCCTA_104
A-EACT-MCAC_082
A-EACT-MCAT_116
A-EACT-MCAC_216
A-EACG-MCTG_206
A-EAAG-MCCAC_112
A-EAAC-MCCATC_294m
A-EAAC-MCCAC_054m
A-EACT-MCAC_105
A-EACT-MCAC_237m
A-EACT-MCCAA_217m
A-EAAG-MCCAC_079
A-EACA-MCTA_101m
SL4-MCTT_190m
A-EAAG-MCCATC_067m
A-EAAG-MCCAC_311
A-EAAG-MCCAC_106
A-EACG-MCCAGC_161m
A-EAAG-MCCAA_106
A-EACT-MCTA_152
A-EAAC-MCCAC_057m
A-EAAC-MCCATC_350
RPMSA_09a
SL4-MCCG_315
SL4-MCCG_507
A-EAAC-MCCAC_104
A-EACG-MCCAG_176
A-EAAG-MCCTA_113
A-EACA-MCTA_105
RPMSG48a
RPMSG45a
RPMSG48b
RPMSG45b
A-EACT-MCCAA_226
A-EAAG-MCCAT_127
A-EAAG-MCCTA_094
A-EAAG-MCCAG_162
A-EACA-MCTA_078
A-EACT-MCCTA_085
A-EAAG-MCCTA_104
A-EAAG-MCCTA_068
A-EACA-MCCACC_146
A-EAAG-MCCTA_116
A-EACT-MCAC_171
A-EAAG-MCCTA_153
A-EACT-MCAC_064
A-EACA-MCCAC_075
A-EAAC-MCCATC_095
A-EACA-MCTA_196
A-EAAC-MCCAG_067
A-EACT-MCAT_095
A-EACT-MCAC_131
A-EAAC-MCCAA_104
A-EAAG-MCCTA_071
A-EAGC-MCTC_395
SL5-MCTT_223
A-EACG-MCCAG_174
A-EACG-MCCAGC_162
A-EAAG-MCCTA_073
A-EACT-MCAC_165
A-EACA-MCCACC_169
A-EACT-MCAC_140
A-EACA-MCAC_079
A-EACT-MCAC_116
A-EACA-MCCACC_189
SL3-MCAC_641
A-EACT-MCTA_109
A-EACT-MCAT_206
A-EAGC-MCTC_434
A-EACT-MCAC_174
A-EACT-MCAC_093
A-EACG-MCCAA_275
SL6-MCCG_068
A-EAAG-MCCTA_067
A-EAAG-MCCAA_249
A-EAAG-MCCATC_110
A-EACT-MCAC_203
A-EAAC-MCCAC_236
A-EACT-MCAC_194
A-EAAC-MCCAG_127
A-EACT-MCTA_097
A-EAAG-MCCATC_240
A-EAGC-MCTC_547
A-EACT-MCAC_062
A-EAAC-MCCAA_109
A-EACT-MCCAA_069
A-EACT-MCAC_266
A-EACG-MCAT_147
A-EAAC-MCCAG_147
A-EACT-MCAC_232
A-EACT-MCAT_088
A-EAGC-MCTC_268
A-EAAG-MCCAG_112
A-EACA-MCCACC_173
A-EACT-MCAT_197
A-EAAG-MCCAT_106
A-EACT-MCAC_397
A-EAAG-MCCAA_115
A-EAGC-MCTG_520
A-EAAC-MCCAC_117
A-EACT-MCCTA_071
SL3-MCAC_308
A-EACG-MCCTA_104
A-EACT-MCAC_082
A-EACT-MCAT_116
A-EACT-MCAC_216
A-EACG-MCTG_206
A-EAAG-MCCAC_112
A-EAAC-MCCATC_294
A-EACA-MCTA_159
A-EAAC-MCCAC_054
A-EACT-MCAC_105
A-EACT-MCAC_171
A-EAAC-MCCAA_169
A-EACT-MCAC_237
A-EAAG-MCCATC_082
A-EACT-MCCAA_217
A-EAAG-MCCAC_079
A-EACA-MCTA_101
SL4-MCTT_190
A-EAAG-MCCATC_067
A-EACG-MCTC_271
A-EACA-MCCAT_056
A-EAAG-MCCAC_311
A-EAAG-MCCAC_106
A-EACG-MCCAGC_161
A-EAAG-MCCAA_106
A-EACT-MCTA_152
A-EAAC-MCCAC_057
A-EAAC-MCCATC_350p
RPMSA09a
SL4-MCCG_315
SL4-MCCG_507p
A-EAAC-MCCAC_104p
A-EACG-MCCAG_176p
A-EAAG-MCCTA_113
A-EACA-MCTA_105
A-EACT-MCCAA_226p
A-EAAG-MCCAT_127
A-EAAG-MCCTA_094
A-EAAG-MCCAG_162
A-EAAG-MCCTA_104p
A-EAAG-MCCTA_068
A-EACA-MCCACC_146p
A-EAAG-MCCTA_116
A-EAAG-MCCTA_153
A-EACA-MCCAC_075
A-EAAC-MCCATC_095
A-EACA-MCTA_196p
A-EACT-MCAT_095
A-EACT-MCAC_131
A-EAAG-MCCTA_071
SL5-MCTT_223p
A-EACG-MCCAG_174p
A-EACG-MCCAGC_162p
A-EAAG-MCCTA_073
A-EACA-MCCACC_169p
A-EACT-MCAC_140
A-EACA-MCCACC_189p
SL3-MCAC_641
A-EACT-MCTA_109
A-EAGC-MCTC_434
A-EACT-MCAC_174
A-EACT-MCAC_093
SL6-MCCG_068p
A-EAAG-MCCTA_067
A-EAAG-MCCAA_249
A-EAAC-MCCAC_236
A-EACT-MCAC_194
A-EACT-MCTA_097
A-EAAG-MCCATC_240p
A-EAGC-MCTC_547
A-EACT-MCCAA_069p
A-EACG-MCAT_147p
A-EAAC-MCCAG_147p
A-EACT-MCAC_232
A-EACT-MCAT_088
A-EAGC-MCTC_268
A-EACA-MCCACC_173p
A-EAAG-MCCAT_106p
A-EAAG-MCCAA_115p
A-EAAC-MCCAC_117
A-EACT-MCCTA_071p
SL3-MCAC_308
A-EACG-MCCTA_104
A-EACT-MCAC_082
A-EACT-MCAT_116
A-EACT-MCAC_216
A-EACG-MCTG_206
A-EAAG-MCCAC_112
A-EACA-MCTA_159p
A-EACT-MCAC_105
A-EACT-MCAC_171p
A-EAAC-MCCAA_169p
A-EAAG-MCCATC_082p
A-EAAG-MCCAC_079
A-EACG-MCTC_271p
A-EACA-MCCAT_056p
A-EAAG-MCCAC_311
A-EAAG-MCCAC_106
A-EAAG-MCCAA_106
A-EACT-MCTA_152

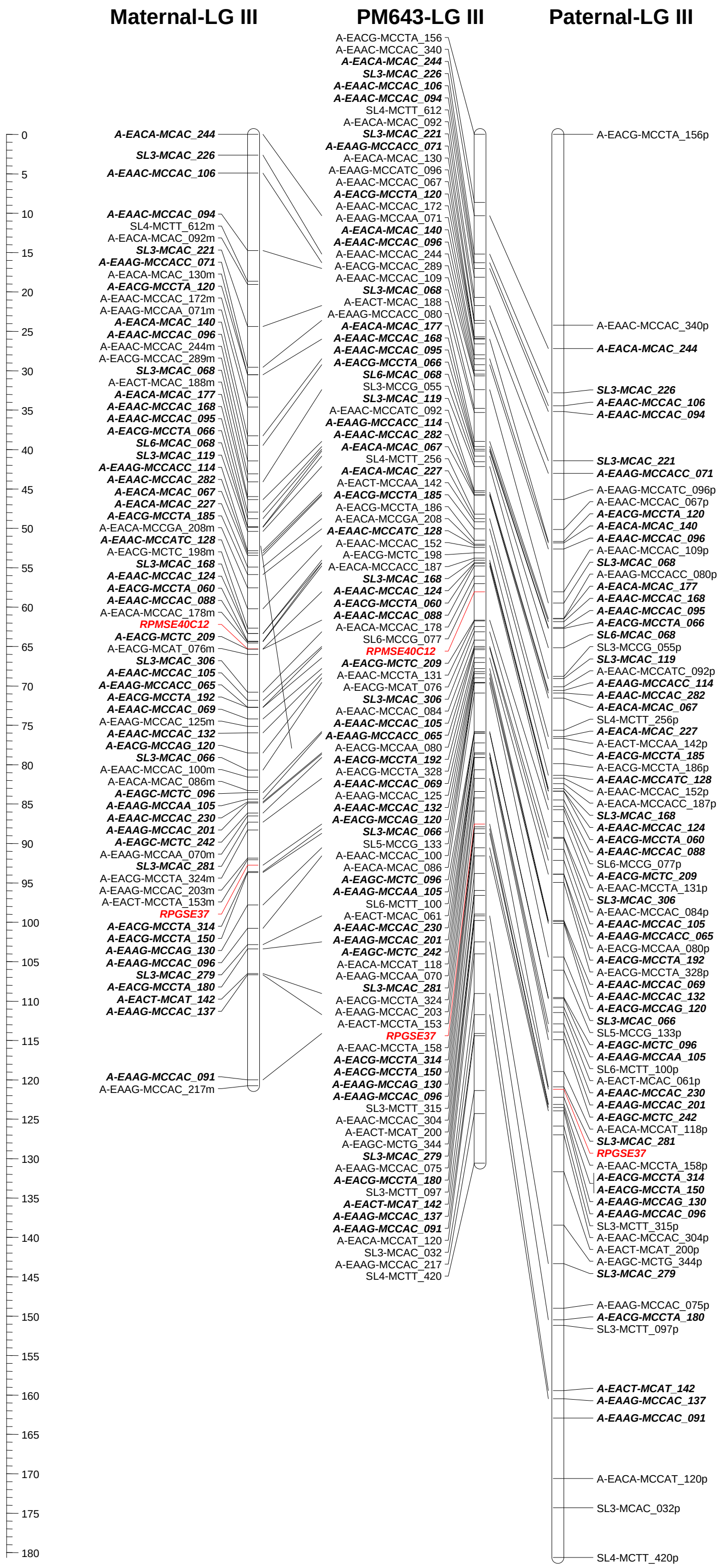

Maternal-LG III
PM643-LG III
Paternal-LG III
RPMSE40C12
RPGSE37

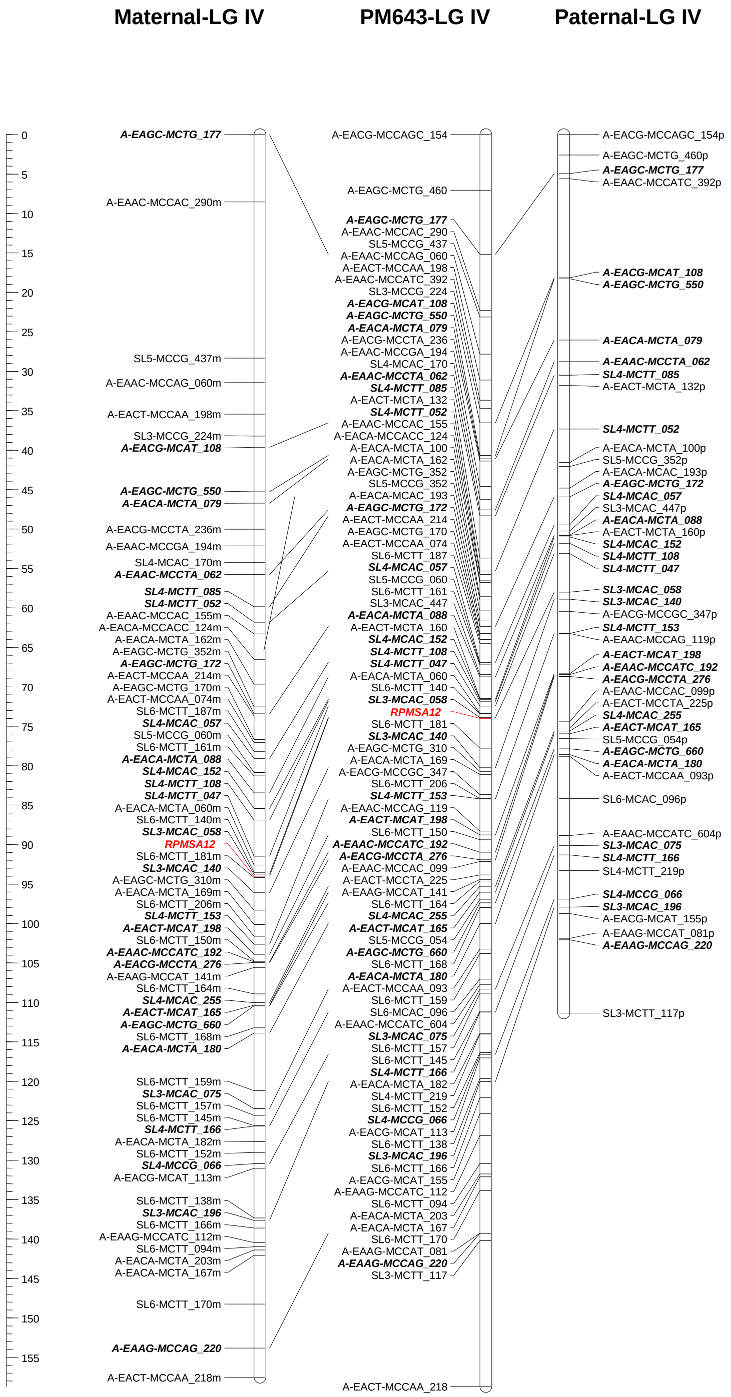

Maternal-LG IV
PM643-LG IV
Paternal-LG IV
0
5
10
15
20
25
30
35
40
45
50
55
60
65
70
75
80
85
90
95
100
105
110
115
120
125
130
135
140
145
150
155
A-EAGC-MCTG_177
A-EAAC-MCCAC_290m
SL5-MCCG_437m
A-EAAC-MCCAG_060m
A-EACT-MCCAA_198m
SL3-MCCG_224m
A-EACG-MCAT_108
A-EAGC-MCTG_550
A-EACA-MCTA_079
A-EACG-MCCTA_236m
A-EAAC-MCCGA_194m
SL4-MCAC_170m
A-EAAC-MCCTA_062
SL4-MCTT_085
SL4-MCTT_052
A-EAAC-MCCAC_155m
A-EACA-MCCACC_124m
A-EACA-MCTA_162m
A-EAGC-MCTG_352m
A-EAGC-MCTG_172
A-EACT-MCCAA_214m
A-EAGC-MCTG_170m
A-EACT-MCCAA_074m
SL6-MCTT_187m
SL4-MCAC_057
SL5-MCCG_060m
SL6-MCTT_161m
A-EACA-MCTA_088
SL4-MCAC_152
SL4-MCTT_108
SL4-MCTT_047
A-EACA-MCTA_060m
SL6-MCTT_140m
SL3-MCAC_058
RPMSA12
SL6-MCTT_181m
SL3-MCAC_140
A-EAGC-MCTG_310m
A-EACA-MCTA_169m
SL6-MCTT_206m
SL4-MCTT_153
A-EACT-MCAT_198
SL6-MCTT_150m
A-EAAC-MCCATC_192
A-EACG-MCCTA_276
A-EAAG-MCCAT_141m
SL6-MCTT_164m
SL4-MCAC_255
A-EACT-MCAT_165
A-EAGC-MCTG_660
SL6-MCTT_168m
A-EACA-MCTA_180
SL6-MCTT_159m
SL3-MCAC_075
SL6-MCTT_157m
SL6-MCTT_145m
SL4-MCTT_166
A-EACA-MCTA_182m
SL6-MCTT_152m
SL4-MCCG_066
A-EACG-MCAT_113m
SL6-MCTT_138m
SL3-MCAC_196
SL6-MCTT_166m
A-EAAG-MCCATC_112m
SL6-MCTT_094m
A-EACA-MCTA_203m
A-EACA-MCTA_167m
SL6-MCTT_170m
A-EAAG-MCCAG_220
A-EACT-MCCAA_218m
A-EACG-MCCAGC_154
A-EAGC-MCTG_460
A-EAGC-MCTG_177
A-EAAC-MCCAC_290
SL5-MCCG_437
A-EAAC-MCCAG_060
A-EACT-MCCAA_198
A-EAAC-MCCATC_392
SL3-MCCG_224
A-EACG-MCAT_108
A-EAGC-MCTG_550
A-EACA-MCTA_079
A-EACG-MCCTA_236
A-EAAC-MCCGA_194
SL4-MCAC_170
A-EAAC-MCCTA_062
SL4-MCTT_085
A-EACT-MCTA_132
SL4-MCTT_052
A-EAAC-MCCAC_155
A-EACA-MCCACC_124
A-EACA-MCTA_100
A-EACA-MCTA_162
A-EAGC-MCTG_352
SL5-MCCG_352
A-EACA-MCAC_193
A-EAGC-MCTG_172
A-EACT-MCCAA_214
A-EAGC-MCTG_170
A-EACT-MCCAA_074
SL6-MCTT_187
SL4-MCAC_057
SL5-MCCG_060
SL6-MCTT_161
SL3-MCAC_447
A-EACA-MCTA_088
A-EACT-MCTA_160
SL4-MCAC_152
SL4-MCTT_108
SL4-MCTT_047
A-EACA-MCTA_060
SL6-MCTT_140
SL3-MCAC_058
RPMSA12
SL6-MCTT_181
SL3-MCAC_140
A-EAGC-MCTG_310
A-EACA-MCTA_169
A-EACG-MCCGC_347
SL6-MCTT_206
SL4-MCTT_153
A-EAAC-MCCAG_119
A-EACT-MCAT_198
SL6-MCTT_150
A-EAAC-MCCATC_192
A-EACG-MCCTA_276
A-EAAC-MCCAC_099
A-EACT-MCCTA_225
A-EAAG-MCCAT_141
SL6-MCTT_164
SL4-MCAC_255
A-EACT-MCAT_165
SL5-MCCG_054
A-EAGC-MCTG_660
SL6-MCTT_168
A-EACA-MCTA_180
A-EACT-MCCAA_093
SL6-MCTT_159
SL6-MCAC_096
A-EAAC-MCCATC_604
SL3-MCAC_075
SL6-MCTT_157
SL6-MCTT_145
SL4-MCTT_166
A-EACA-MCTA_182
SL4-MCTT_219
SL6-MCTT_152
SL4-MCCG_066
A-EACG-MCAT_113
SL6-MCTT_138
SL3-MCAC_196
SL6-MCTT_166
A-EACG-MCAT_155
A-EAAG-MCCATC_112
SL6-MCTT_094
A-EACA-MCTA_203
A-EACA-MCTA_167
SL6-MCTT_170
A-EAAG-MCCAT_081
A-EAAG-MCCAG_220
SL3-MCTT_117
A-EACT-MCCAA_218
A-EACG-MCCAGC_154p
A-EAGC-MCTG_460p
A-EAGC-MCTG_177
A-EAAC-MCCATC_392p
A-EACG-MCAT_108
A-EAGC-MCTG_550
A-EACA-MCTA_079
A-EAAC-MCCTA_062
SL4-MCTT_085
A-EACT-MCTA_132p
SL4-MCTT_052
A-EACA-MCTA_100p
SL5-MCCG_352p
A-EACA-MCAC_193p
A-EAGC-MCTG_172
SL4-MCAC_057
SL3-MCAC_447p
A-EACA-MCTA_088
A-EACT-MCTA_160p
SL4-MCAC_152
SL4-MCTT_108
SL4-MCTT_047
SL3-MCAC_058
SL3-MCAC_140
A-EACG-MCCGC_347p
SL4-MCTT_153
A-EAAC-MCCAG_119p
A-EACT-MCAT_198
A-EAAC-MCCATC_192
A-EACG-MCCTA_276
A-EAAC-MCCAC_099p
A-EACT-MCCTA_225p
SL4-MCAC_255
A-EACT-MCAT_165
SL5-MCCG_054p
A-EAGC-MCTG_660
A-EACA-MCTA_180
A-EACT-MCCAA_093p
SL6-MCAC_096p
A-EAAC-MCCATC_604p
SL3-MCAC_075
SL4-MCTT_166
SL4-MCTT_219p
SL4-MCCG_066
SL3-MCAC_196
A-EACG-MCAT_155p
A-EAAG-MCCAT_081p
A-EAAG-MCCAG_220
SL3-MCTT_117p

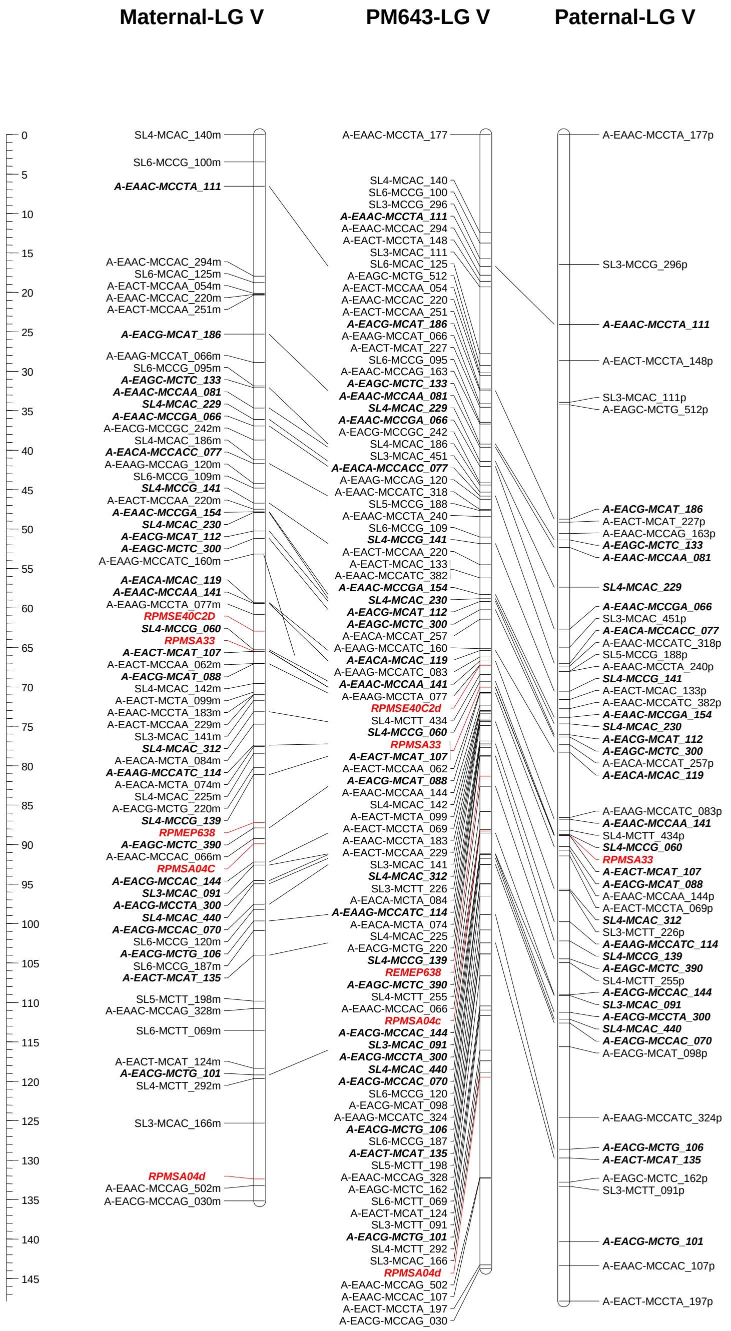

Maternal-LG V
PM643-LG V
Paternal-LG V
0
5
10
15
20
25
30
35
40
45
50
55
60
65
70
75
80
85
90
95
100
105
110
115
120
125
130
135
140
145
SL4-MCAC_140m
SL6-MCCG_100m
A-EAAC-MCCTA_111
A-EAAC-MCCAC_294m
SL6-MCAC_125m
A-EACT-MCCAA_054m
A-EAAC-MCCAC_220m
A-EACT-MCCAA_251m
A-EACG-MCAT_186
A-EAAG-MCCAT_066m
SL6-MCCG_095m
A-EAGC-MCTC_133
A-EAAC-MCCAA_081
SL4-MCAC_229
A-EAAC-MCCGA_066
A-EACG-MCCGC_242m
SL4-MCAC_186m
A-EACA-MCCACC_077
A-EAAG-MCCAG_120m
SL6-MCCG_109m
SL4-MCCG_141
A-EACT-MCCAA_220m
A-EAAC-MCCGA_154
SL4-MCAC_230
A-EACG-MCAT_112
A-EAGC-MCTC_300
A-EAAG-MCCATC_160m
A-EACA-MCAC_119
A-EAAC-MCCAA_141
A-EAAG-MCCTA_077m
RPMSE40C2D
SL4-MCCG_060
RPMSA33
A-EACT-MCAT_107
A-EACT-MCCAA_062m
A-EACG-MCAT_088
SL4-MCAC_142m
A-EACT-MCTA_099m
A-EAAC-MCCTA_183m
A-EACT-MCCAA_229m
SL3-MCAC_141m
SL4-MCAC_312
A-EACA-MCTA_084m
A-EAAG-MCCATC_114
A-EACA-MCTA_074m
SL4-MCAC_225m
A-EACG-MCTG_220m
SL4-MCCG_139
RPMEP638
A-EAGC-MCTC_390
A-EAAC-MCCAC_066m
RPMSA04C
A-EACG-MCCAC_144
SL3-MCAC_091
A-EACG-MCCTA_300
SL4-MCAC_440
A-EACG-MCCAC_070
SL6-MCCG_120m
A-EACG-MCTG_106
SL6-MCCG_187m
A-EACT-MCAT_135
SL5-MCTT_198m
A-EAAC-MCCAG_328m
SL6-MCTT_069m
A-EACT-MCAT_124m
A-EACG-MCTG_101
SL4-MCTT_292m
SL3-MCAC_166m
RPMSA04d
A-EAAC-MCCAG_502m
A-EACG-MCCAG_030m
A-EAAC-MCCTA_177
SL4-MCAC_140
SL6-MCCG_100
SL3-MCCG_296
A-EAAC-MCCTA_111
A-EAAC-MCCAC_294
A-EACT-MCCTA_148
SL3-MCAC_111
SL6-MCAC_125
A-EAGC-MCTG_512
A-EACT-MCCAA_054
A-EAAC-MCCAC_220
A-EACT-MCCAA_251
A-EACG-MCAT_186
A-EAAG-MCCAT_066
A-EACT-MCAT_227
SL6-MCCG_095
A-EAAC-MCCAG_163
A-EAGC-MCTC_133
A-EAAC-MCCAA_081
SL4-MCAC_229
A-EAAC-MCCGA_066
A-EACG-MCCGC_242
SL4-MCAC_186
SL3-MCAC_451
A-EACA-MCCACC_077
A-EAAG-MCCAG_120
A-EAAC-MCCATC_318
SL5-MCCG_188
A-EAAC-MCCTA_240
SL6-MCCG_109
SL4-MCCG_141
A-EACT-MCCAA_220
A-EACT-MCAC_133
A-EAAC-MCCATC_382
A-EAAC-MCCGA_154
SL4-MCAC_230
A-EACG-MCAT_112
A-EAGC-MCTC_300
A-EACA-MCCAT_257
A-EAAG-MCCATC_160
A-EACA-MCAC_119
A-EAAG-MCCATC_083
A-EAAC-MCCAA_141
A-EAAG-MCCTA_077
RPMSE40C2d
SL4-MCTT_434
SL4-MCCG_060
RPMSA33
A-EACT-MCAT_107
A-EACT-MCCAA_062
A-EACG-MCAT_088
A-EAAC-MCCAA_144
SL4-MCAC_142
A-EACT-MCTA_099
A-EACT-MCCTA_069
A-EAAC-MCCTA_183
A-EACT-MCCAA_229
SL3-MCAC_141
SL4-MCAC_312
SL3-MCTT_226
A-EACA-MCTA_084
A-EAAG-MCCATC_114
A-EACA-MCTA_074
SL4-MCAC_225
A-EACG-MCTG_220
SL4-MCCG_139
REMEP638
A-EAGC-MCTC_390
SL4-MCTT_255
A-EAAC-MCCAC_066
RPMSA04c
A-EACG-MCCAC_144
SL3-MCAC_091
A-EACG-MCCTA_300
SL4-MCAC_440
A-EACG-MCCAC_070
SL6-MCCG_120
A-EACG-MCAT_098
A-EAAG-MCCATC_324
A-EACG-MCTG_106
SL6-MCCG_187
A-EACT-MCAT_135
SL5-MCTT_198
A-EAAC-MCCAG_328
A-EAGC-MCTC_162
SL6-MCTT_069
A-EACT-MCAT_124
SL3-MCTT_091
A-EACG-MCTG_101
SL4-MCTT_292
SL3-MCAC_166
RPMSA04d
A-EAAC-MCCAG_502
A-EAAC-MCCAC_107
A-EACT-MCCTA_197
A-EACG-MCCAG_030
A-EAAC-MCCTA_177p
SL3-MCCG_296p
A-EAAC-MCCTA_111
A-EACT-MCCTA_148p
SL3-MCAC_111p
A-EAGC-MCTG_512p
A-EACG-MCAT_186
A-EACT-MCAT_227p
A-EAAC-MCCAG_163p
A-EAGC-MCTC_133
A-EAAC-MCCAA_081
SL4-MCAC_229
A-EAAC-MCCGA_066
SL3-MCAC_451p
A-EACA-MCCACC_077
A-EAAC-MCCATC_318p
SL5-MCCG_188p
A-EAAC-MCCTA_240p
SL4-MCCG_141
A-EACT-MCAC_133p
A-EAAC-MCCATC_382p
A-EAAC-MCCGA_154
SL4-MCAC_230
A-EACG-MCAT_112
A-EAGC-MCTC_300
A-EACA-MCCAT_257p
A-EACA-MCAC_119
A-EAAG-MCCATC_083p
A-EAAC-MCCAA_141
SL4-MCTT_434p
SL4-MCCG_060
RPMSA33
A-EACT-MCAT_107
A-EACG-MCAT_088
A-EAAC-MCCAA_144p
A-EACT-MCCTA_069p
SL4-MCAC_312
SL3-MCTT_226p
A-EAAG-MCCATC_114
SL4-MCCG_139
A-EAGC-MCTC_390
SL4-MCTT_255p
A-EACG-MCCAC_144
SL3-MCAC_091
A-EACG-MCCTA_300
SL4-MCAC_440
A-EACG-MCCAC_070
A-EACG-MCAT_098p
A-EAAG-MCCATC_324p
A-EACG-MCTG_106
A-EACT-MCAT_135
A-EAGC-MCTC_162p
SL3-MCTT_091p
A-EACG-MCTG_101
A-EAAC-MCCAC_107p
A-EACT-MCCTA_197p

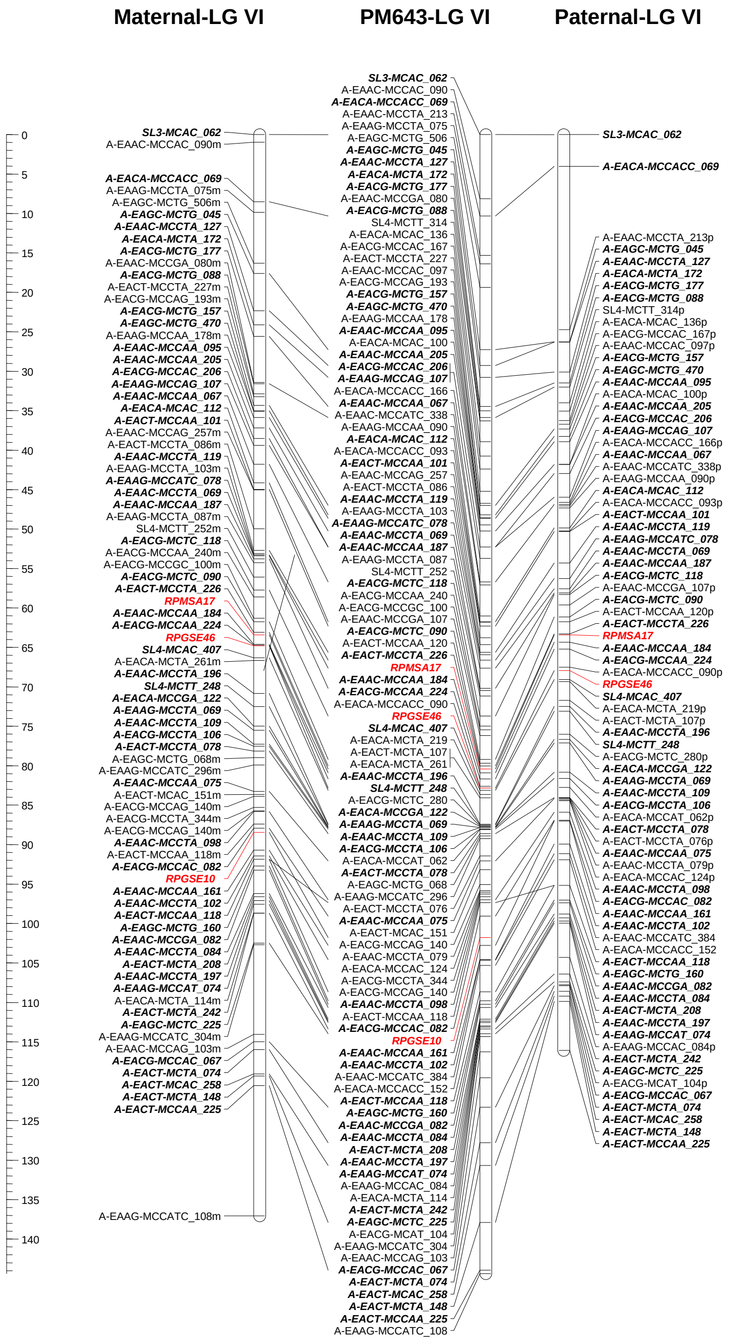
Maternal-LG VI
PM643-LG VI
Paternal-LG VI
0
5
10
15
20
25
30
35
40
45
50
55
60
65
70
75
80
85
90
95
100
105
110
115
120
125
130
135
140
SL3-MCAC_062
A-EAAC-MCCAC_090m
A-EACA-MCCACC_069
A-EAAG-MCCTA_075m
A-EAGC-MCTG_506m
A-EAGC-MCTG_045
A-EAAC-MCCTA_127
A-EACA-MCTA_172
A-EACG-MCTG_177
A-EAAC-MCCGA_080m
A-EACG-MCTG_088
A-EACT-MCCTA_227m
A-EACG-MCCAG_193m
A-EACG-MCTG_157
A-EAGC-MCTG_470
A-EAAG-MCCAA_178m
A-EAAC-MCCAA_095
A-EAAC-MCCAA_205
A-EACG-MCCAC_206
A-EAAG-MCCAG_107
A-EAAC-MCCAA_067
A-EACA-MCAC_112
A-EACT-MCCAA_101
A-EAAC-MCCAG_257m
A-EACT-MCCTA_086m
A-EAAC-MCCTA_119
A-EAAG-MCCTA_103m
A-EAAG-MCCATC_078
A-EAAC-MCCTA_069
A-EAAC-MCCAA_187
A-EAAG-MCCTA_087m
SL4-MCTT_252m
A-EACG-MCTC_118
A-EACG-MCCAA_240m
A-EACG-MCCGC_100m
A-EACG-MCTC_090
A-EACT-MCCTA_226
RPMSA17
A-EAAC-MCCAA_184
A-EACG-MCCAA_224
RPGSE46
SL4-MCAC_407
A-EACA-MCTA_261m
A-EAAC-MCCTA_196
SL4-MCTT_248
A-EACA-MCCGA_122
A-EAAG-MCCTA_069
A-EAAC-MCCTA_109
A-EACG-MCCTA_106
A-EACT-MCCTA_078
A-EAGC-MCTG_068m
A-EAAG-MCCATC_296m
A-EAAC-MCCAA_075
A-EACT-MCAC_151m
A-EACG-MCCAG_140m
A-EACG-MCCTA_344m
A-EACG-MCCAG_140m
A-EAAC-MCCTA_098
A-EACT-MCCAA_118m
A-EACG-MCCAC_082
RPGSE10
A-EAAC-MCCAA_161
A-EAAC-MCCTA_102
A-EACT-MCCAA_118
A-EAGC-MCTG_160
A-EAAC-MCCGA_082
A-EAAC-MCCTA_084
A-EACT-MCTA_208
A-EAAC-MCCTA_197
A-EAAG-MCCAT_074
A-EACA-MCTA_114m
A-EACT-MCTA_242
A-EAGC-MCTC_225
A-EAAG-MCCATC_304m
A-EAAC-MCCAG_103m
A-EACG-MCCAC_067
A-EACT-MCTA_074
A-EACT-MCAC_258
A-EACT-MCTA_148
A-EACT-MCCAA_225
A-EAAG-MCCATC_108m
SL3-MCAC_062
A-EAAC-MCCAC_090
A-EACA-MCCACC_069
A-EAAC-MCCTA_213
A-EAAG-MCCTA_075
A-EAGC-MCTG_506
A-EAGC-MCTG_045
A-EAAC-MCCTA_127
A-EACA-MCTA_172
A-EACG-MCTG_177
A-EAAC-MCCGA_080
A-EACG-MCTG_088
SL4-MCTT_314
A-EACA-MCAC_136
A-EACG-MCCAC_167
A-EACT-MCCTA_227
A-EAAC-MCCAC_097
A-EACG-MCCAG_193
A-EACG-MCTG_157
A-EAGC-MCTG_470
A-EAAG-MCCAA_178
A-EAAC-MCCAA_095
A-EACA-MCAC_100
A-EAAC-MCCAA_205
A-EACG-MCCAC_206
A-EAAG-MCCAG_107
A-EACA-MCCACC_166
A-EAAC-MCCAA_067
A-EAAC-MCCATC_338
A-EAAG-MCCAA_090
A-EACA-MCAC_112
A-EACA-MCCACC_093
A-EACT-MCCAA_101
A-EAAC-MCCAG_257
A-EACT-MCCTA_086
A-EAAC-MCCTA_119
A-EAAG-MCCTA_103
A-EAAG-MCCATC_078
A-EAAC-MCCTA_069
A-EAAC-MCCAA_187
A-EAAG-MCCTA_087
SL4-MCTT_252
A-EACG-MCTC_118
A-EACG-MCCAA_240
A-EACG-MCCGC_100
A-EAAC-MCCGA_107
A-EACG-MCTC_090
A-EACT-MCCAA_120
A-EACT-MCCTA_226
RPMSA17
A-EAAC-MCCAA_184
A-EACG-MCCAA_224
A-EACA-MCCACC_090
RPGSE46
SL4-MCAC_407
A-EACA-MCTA_219
A-EACT-MCTA_107
A-EACA-MCTA_261
A-EAAC-MCCTA_196
SL4-MCTT_248
A-EACG-MCTC_280
A-EACA-MCCGA_122
A-EAAG-MCCTA_069
A-EAAC-MCCTA_109
A-EACG-MCCTA_106
A-EACA-MCCAT_062
A-EACT-MCCTA_078
A-EAGC-MCTG_068
A-EAAG-MCCATC_296
A-EACT-MCCTA_076
A-EAAC-MCCAA_075
A-EACT-MCAC_151
A-EACG-MCCAG_140
A-EAAC-MCCTA_079
A-EACA-MCCAC_124
A-EACG-MCCTA_344
A-EACG-MCCAG_140
A-EAAC-MCCTA_098
A-EACT-MCCAA_118
A-EACG-MCCAC_082
RPGSE10
A-EAAC-MCCAA_161
A-EAAC-MCCTA_102
A-EAAC-MCCATC_384
A-EACA-MCCACC_152
A-EACT-MCCAA_118
A-EAGC-MCTG_160
A-EAAC-MCCGA_082
A-EAAC-MCCTA_084
A-EACT-MCTA_208
A-EAAC-MCCTA_197
A-EAAG-MCCAT_074
A-EAAG-MCCAC_084
A-EACA-MCTA_114
A-EACT-MCTA_242
A-EAGC-MCTC_225
A-EACG-MCAT_104
A-EAAG-MCCATC_304
A-EAAC-MCCAG_103
A-EACG-MCCAC_067
A-EACT-MCTA_074
A-EACT-MCAC_258
A-EACT-MCTA_148
A-EACT-MCCAA_225
A-EAAG-MCCATC_108
SL3-MCAC_062
A-EACA-MCCACC_069
A-EAAC-MCCTA_213p
A-EAGC-MCTG_045
A-EAAC-MCCTA_127
A-EACA-MCTA_172
A-EACG-MCTG_177
A-EACG-MCTG_088
SL4-MCTT_314p
A-EACA-MCAC_136p
A-EACG-MCCAC_167p
A-EAAC-MCCAC_097p
A-EACG-MCTG_157
A-EAGC-MCTG_470
A-EAAC-MCCAA_095
A-EACA-MCAC_100p
A-EAAC-MCCAA_205
A-EACG-MCCAC_206
A-EAAG-MCCAG_107
A-EACA-MCCACC_166p
A-EAAC-MCCAA_067
A-EAAC-MCCATC_338p
A-EAAG-MCCAA_090p
A-EACA-MCAC_112
A-EACA-MCCACC_093p
A-EACT-MCCAA_101
A-EAAC-MCCTA_119
A-EAAG-MCCATC_078
A-EAAC-MCCTA_069
A-EAAC-MCCAA_187
A-EACG-MCTC_118
A-EAAC-MCCGA_107p
A-EACG-MCTC_090
A-EACT-MCCAA_120p
A-EACT-MCCTA_226
RPMSA17
A-EAAC-MCCAA_184
A-EACG-MCCAA_224
A-EACA-MCCACC_090p
RPGSE46
SL4-MCAC_407
A-EACA-MCTA_219p
A-EACT-MCTA_107p
A-EAAC-MCCTA_196
SL4-MCTT_248
A-EACG-MCTC_280p
A-EACA-MCCGA_122
A-EAAG-MCCTA_069
A-EAAC-MCCTA_109
A-EACG-MCCTA_106
A-EACA-MCCAT_062p
A-EACT-MCCTA_078
A-EACT-MCCTA_076p
A-EAAC-MCCAA_075
A-EAAC-MCCTA_079p
A-EACA-MCCAC_124p
A-EAAC-MCCTA_098
A-EACG-MCCAC_082
A-EAAC-MCCAA_161
A-EAAC-MCCTA_102
A-EAAC-MCCATC_384
A-EACA-MCCACC_152
A-EACT-MCCAA_118
A-EAGC-MCTG_160
A-EAAC-MCCGA_082
A-EAAC-MCCTA_084
A-EACT-MCTA_208
A-EAAC-MCCTA_197
A-EAAG-MCCAT_074
A-EAAG-MCCAC_084p
A-EACT-MCTA_242
A-EAGC-MCTC_225
A-EACG-MCAT_104p
A-EACG-MCCAC_067
A-EACT-MCTA_074
A-EACT-MCAC_258
A-EACT-MCTA_148
A-EACT-MCCAA_225

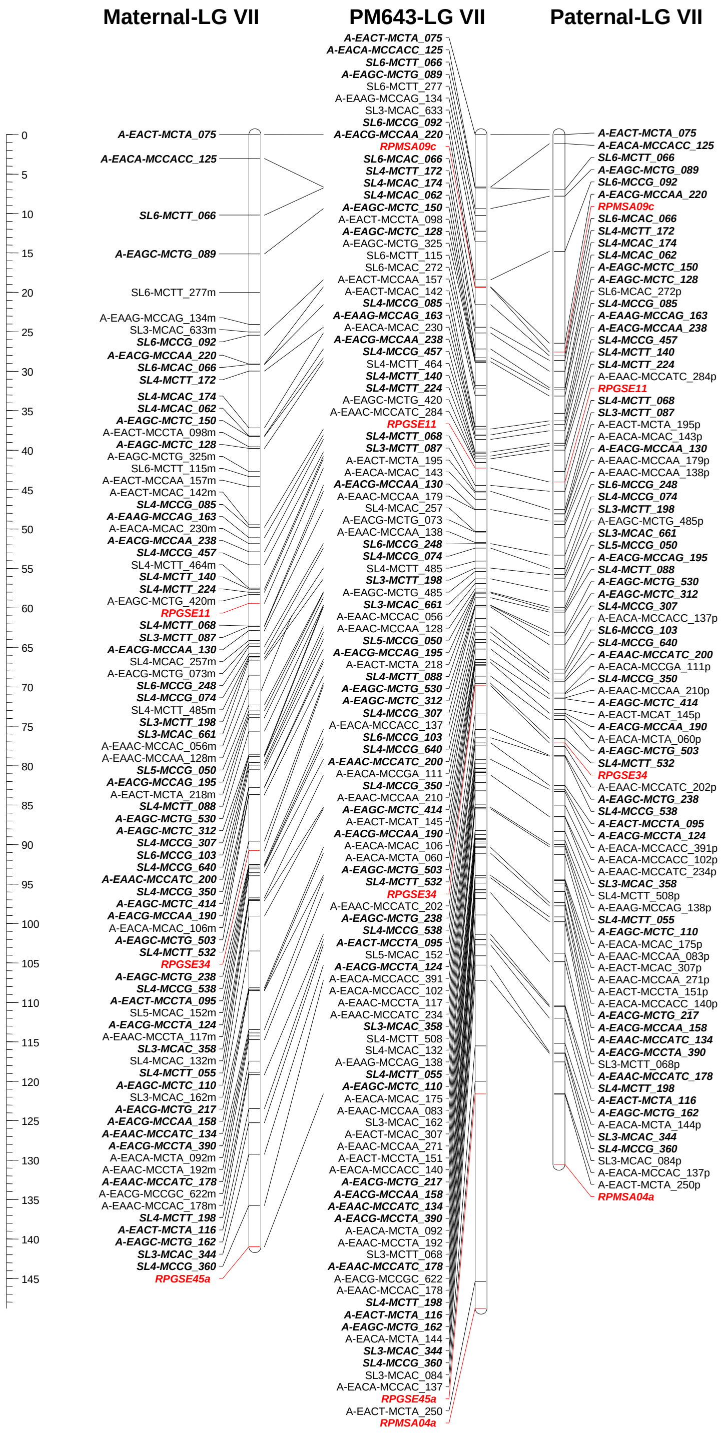

Maternal-LG VII
PM643-LG VII
Paternal-LG VII
0
5
10
15
20
25
30
35
40
45
50
55
60
65
70
75
80
85
90
95
100
105
110
115
120
125
130
135
140
145
A-EACT-MCTA_075
A-EACA-MCCACC_125
SL6-MCTT_066
A-EAGC-MCTG_089
SL6-MCTT_277m
A-EAAG-MCCAG_134m
SL3-MCAC_633m
SL6-MCCG_092
A-EACG-MCCAA_220
SL6-MCAC_066
SL4-MCTT_172
SL4-MCAC_174
SL4-MCAC_062
A-EAGC-MCTC_150
A-EACT-MCCTA_098m
A-EAGC-MCTC_128
A-EAGC-MCTG_325m
SL6-MCTT_115m
A-EACT-MCCAA_157m
A-EACT-MCAC_142m
SL4-MCCG_085
A-EAAG-MCCAG_163
A-EACA-MCAC_230m
A-EACG-MCCAA_238
SL4-MCCG_457
SL4-MCTT_464m
SL4-MCTT_140
SL4-MCTT_224
A-EAGC-MCTG_420m
RPGSE11
SL4-MCTT_068
SL3-MCTT_087
A-EACG-MCCAA_130
SL4-MCAC_257m
A-EACG-MCTG_073m
SL6-MCCG_248
SL4-MCCG_074
SL4-MCTT_485m
SL3-MCTT_198
SL3-MCAC_661
A-EAAC-MCCAC_056m
A-EAAC-MCCAA_128m
SL5-MCCG_050
A-EACG-MCCAG_195
A-EACT-MCTA_218m
SL4-MCTT_088
A-EAGC-MCTG_530
A-EAGC-MCTC_312
SL4-MCCG_307
SL6-MCCG_103
SL4-MCCG_640
A-EAAC-MCCATC_200
SL4-MCCG_350
A-EAGC-MCTC_414
A-EACG-MCCAA_190
A-EACA-MCAC_106m
A-EAGC-MCTG_503
SL4-MCTT_532
RPGSE34
A-EAGC-MCTG_238
SL4-MCCG_538
A-EACT-MCCTA_095
SL5-MCAC_152m
A-EACG-MCCTA_124
A-EAAC-MCCTA_117m
SL3-MCAC_358
SL4-MCAC_132m
SL4-MCTT_055
A-EAGC-MCTC_110
SL3-MCAC_162m
A-EACG-MCTG_217
A-EACG-MCCAA_158
A-EAAC-MCCATC_134
A-EACG-MCCTA_390
A-EACA-MCTA_092m
A-EAAC-MCCTA_192m
A-EAAC-MCCATC_178
A-EACG-MCCGC_622m
A-EAAC-MCCAC_178m
SL4-MCTT_198
A-EACT-MCTA_116
A-EAGC-MCTG_162
SL3-MCAC_344
SL4-MCCG_360
RPGSE45a
A-EACT-MCTA_075
A-EACA-MCCACC_125
SL6-MCTT_066
A-EAGC-MCTG_089
SL6-MCTT_277
A-EAAG-MCCAG_134
SL3-MCAC_633
SL6-MCCG_092
A-EACG-MCCAA_220
RPMSA09c
SL6-MCAC_066
SL4-MCTT_172
SL4-MCAC_174
SL4-MCAC_062
A-EAGC-MCTC_150
A-EACT-MCCTA_098
A-EAGC-MCTC_128
A-EAGC-MCTG_325
SL6-MCTT_115
SL6-MCAC_272
A-EACT-MCCAA_157
A-EACT-MCAC_142
SL4-MCCG_085
A-EAAG-MCCAG_163
A-EACA-MCAC_230
A-EACG-MCCAA_238
SL4-MCCG_457
SL4-MCTT_464
SL4-MCTT_140
SL4-MCTT_224
A-EAGC-MCTG_420
A-EAAC-MCCATC_284
RPGSE11
SL4-MCTT_068
SL3-MCTT_087
A-EACT-MCTA_195
A-EACA-MCAC_143
A-EACG-MCCAA_130
A-EAAC-MCCAA_179
SL4-MCAC_257
A-EACG-MCTG_073
A-EAAC-MCCAA_138
SL6-MCCG_248
SL4-MCCG_074
SL4-MCTT_485
SL3-MCTT_198
A-EAGC-MCTG_485
SL3-MCAC_661
A-EAAC-MCCAC_056
A-EAAC-MCCAA_128
SL5-MCCG_050
A-EACG-MCCAG_195
A-EACT-MCTA_218
SL4-MCTT_088
A-EAGC-MCTG_530
A-EAGC-MCTC_312
SL4-MCCG_307
A-EACA-MCCACC_137
SL6-MCCG_103
SL4-MCCG_640
A-EAAC-MCCATC_200
A-EACA-MCCGA_111
SL4-MCCG_350
A-EAAC-MCCAA_210
A-EAGC-MCTC_414
A-EACT-MCAT_145
A-EACG-MCCAA_190
A-EACA-MCAC_106
A-EACA-MCTA_060
A-EAGC-MCTG_503
SL4-MCTT_532
RPGSE34
A-EAAC-MCCATC_202
A-EAGC-MCTG_238
SL4-MCCG_538
A-EACT-MCCTA_095
SL5-MCAC_152
A-EACG-MCCTA_124
A-EACA-MCCACC_391
A-EACA-MCCACC_102
A-EAAC-MCCTA_117
A-EAAC-MCCATC_234
SL3-MCAC_358
SL4-MCTT_508
SL4-MCAC_132
A-EAAG-MCCAG_138
SL4-MCTT_055
A-EAGC-MCTC_110
A-EACA-MCAC_175
A-EAAC-MCCAA_083
SL3-MCAC_162
A-EACT-MCAC_307
A-EAAC-MCCAA_271
A-EACT-MCCTA_151
A-EACA-MCCACC_140
A-EACG-MCTG_217
A-EACG-MCCAA_158
A-EAAC-MCCATC_134
A-EACG-MCCTA_390
A-EACA-MCTA_092
A-EAAC-MCCTA_192
SL3-MCTT_068
A-EAAC-MCCATC_178
A-EACG-MCCGC_622
A-EAAC-MCCAC_178
SL4-MCTT_198
A-EACT-MCTA_116
A-EAGC-MCTG_162
A-EACA-MCTA_144
SL3-MCAC_344
SL4-MCCG_360
SL3-MCAC_084
A-EACA-MCCAC_137
RPGSE45a
A-EACT-MCTA_250
RPMSA04a
A-EACT-MCTA_075
A-EACA-MCCACC_125
SL6-MCTT_066
A-EAGC-MCTG_089
SL6-MCCG_092
A-EACG-MCCAA_220
RPMSA09c
SL6-MCAC_066
SL4-MCTT_172
SL4-MCAC_174
SL4-MCAC_062
A-EAGC-MCTC_150
A-EAGC-MCTC_128
SL6-MCAC_272p
SL4-MCCG_085
A-EAAG-MCCAG_163
A-EACG-MCCAA_238
SL4-MCCG_457
SL4-MCTT_140
SL4-MCTT_224
A-EAAC-MCCATC_284p
RPGSE11
SL4-MCTT_068
SL3-MCTT_087
A-EACT-MCTA_195p
A-EACA-MCAC_143p
A-EACG-MCCAA_130
A-EAAC-MCCAA_179p
A-EAAC-MCCAA_138p
SL6-MCCG_248
SL4-MCCG_074
SL3-MCTT_198
A-EAGC-MCTG_485p
SL3-MCAC_661
SL5-MCCG_050
A-EACG-MCCAG_195
SL4-MCTT_088
A-EAGC-MCTG_530
A-EAGC-MCTC_312
SL4-MCCG_307
A-EACA-MCCACC_137p
SL6-MCCG_103
SL4-MCCG_640
A-EAAC-MCCATC_200
A-EACA-MCCGA_111p
SL4-MCCG_350
A-EAAC-MCCAA_210p
A-EAGC-MCTC_414
A-EACT-MCAT_145p
A-EACG-MCCAA_190
A-EACA-MCTA_060p
A-EAGC-MCTG_503
SL4-MCTT_532
RPGSE34
A-EAAC-MCCATC_202p
A-EAGC-MCTG_238
SL4-MCCG_538
A-EACT-MCCTA_095
A-EACG-MCCTA_124
A-EACA-MCCACC_391p
A-EACA-MCCACC_102p
A-EAAC-MCCATC_234p
SL3-MCAC_358
SL4-MCTT_508p
A-EAAG-MCCAG_138p
SL4-MCTT_055
A-EAGC-MCTC_110
A-EACA-MCAC_175p
A-EAAC-MCCAA_083p
A-EACT-MCAC_307p
A-EAAC-MCCAA_271p
A-EACT-MCCTA_151p
A-EACA-MCCACC_140p
A-EACG-MCTG_217
A-EACG-MCCAA_158
A-EAAC-MCCATC_134
A-EACG-MCCTA_390
SL3-MCTT_068p
A-EAAC-MCCATC_178
SL4-MCTT_198
A-EACT-MCTA_116
A-EAGC-MCTG_162
A-EACA-MCTA_144p
SL3-MCAC_344
SL4-MCCG_360
SL3-MCAC_084p
A-EACA-MCCAC_137p
A-EACT-MCTA_250p
RPMSA04a

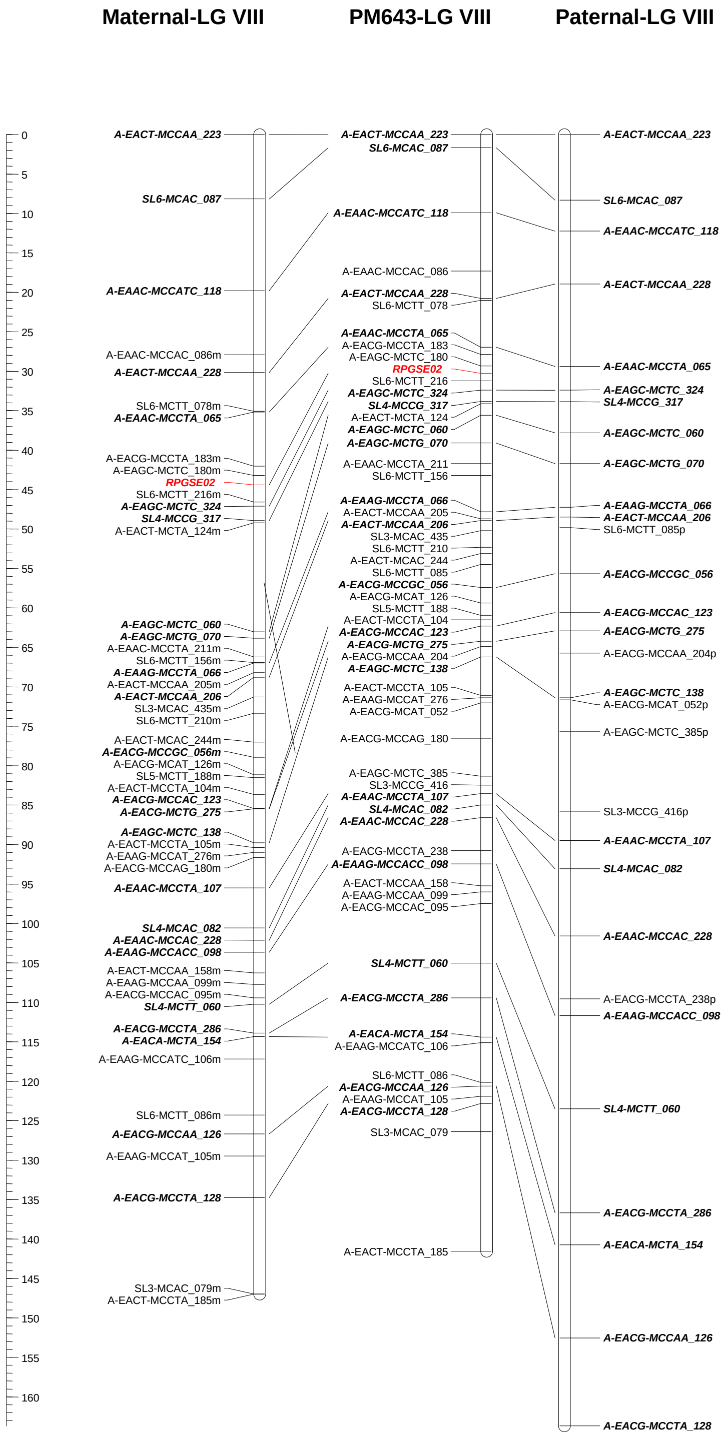

Maternal-LG VIII
PM643-LG VIII
Paternal-LG VIII
0
5
10
15
20
25
30
35
40
45
50
55
60
65
70
75
80
85
90
95
100
105
110
115
120
125
130
135
140
145
150
155
160
A-EACT-MCCAA_223
SL6-MCAC_087
A-EAAC-MCCATC_118
A-EAAC-MCCAC_086m
A-EACT-MCCAA_228
SL6-MCTT_078m
A-EAAC-MCCTA_065
A-EACG-MCCTA_183m
A-EAGC-MCTC_180m
RPGSE02
SL6-MCTT_216m
A-EAGC-MCTC_324
SL4-MCCG_317
A-EACT-MCTA_124m
A-EAGC-MCTC_060
A-EAGC-MCTG_070
A-EAAC-MCCTA_211m
SL6-MCTT_156m
A-EAAG-MCCTA_066
A-EACT-MCCAA_205m
A-EACT-MCCAA_206
SL3-MCAC_435m
SL6-MCTT_210m
A-EACT-MCAC_244m
A-EACG-MCCGC_056m
A-EACG-MCAT_126m
SL5-MCTT_188m
A-EACT-MCCTA_104m
A-EACG-MCCAC_123
A-EACG-MCTG_275
A-EAGC-MCTC_138
A-EACT-MCCTA_105m
A-EAAG-MCCAT_276m
A-EACG-MCCAG_180m
A-EAAC-MCCTA_107
SL4-MCAC_082
A-EAAC-MCCAC_228
A-EAAG-MCCACC_098
A-EACT-MCCAA_158m
A-EAAG-MCCAA_099m
A-EACG-MCCAC_095m
SL4-MCTT_060
A-EACG-MCCTA_286
A-EACA-MCTA_154
A-EAAG-MCCATC_106m
SL6-MCTT_086m
A-EACG-MCCAA_126
A-EAAG-MCCAT_105m
A-EACG-MCCTA_128
SL3-MCAC_079m
A-EACT-MCCTA_185m
A-EACT-MCCAA_223
SL6-MCAC_087
A-EAAC-MCCATC_118
A-EAAC-MCCAC_086
A-EACT-MCCAA_228
SL6-MCTT_078
A-EAAC-MCCTA_065
A-EACG-MCCTA_183
A-EAGC-MCTC_180
RPGSE02
SL6-MCTT_216
A-EAGC-MCTC_324
SL4-MCCG_317
A-EACT-MCTA_124
A-EAGC-MCTC_060
A-EAGC-MCTG_070
A-EAAC-MCCTA_211
SL6-MCTT_156
A-EAAG-MCCTA_066
A-EACT-MCCAA_205
A-EACT-MCCAA_206
SL3-MCAC_435
SL6-MCTT_210
A-EACT-MCAC_244
SL6-MCTT_085
A-EACG-MCCGC_056
A-EACG-MCAT_126
SL5-MCTT_188
A-EACT-MCCTA_104
A-EACG-MCCAC_123
A-EACG-MCTG_275
A-EACG-MCCAA_204
A-EAGC-MCTC_138
A-EACT-MCCTA_105
A-EAAG-MCCAT_276
A-EACG-MCAT_052
A-EACG-MCCAG_180
A-EAGC-MCTC_385
SL3-MCCG_416
A-EAAC-MCCTA_107
SL4-MCAC_082
A-EAAC-MCCAC_228
A-EACG-MCCTA_238
A-EAAG-MCCACC_098
A-EACT-MCCAA_158
A-EAAG-MCCAA_099
A-EACG-MCCAC_095
SL4-MCTT_060
A-EACG-MCCTA_286
A-EACA-MCTA_154
A-EAAG-MCCATC_106
SL6-MCTT_086
A-EACG-MCCAA_126
A-EAAG-MCCAT_105
A-EACG-MCCTA_128
SL3-MCAC_079
A-EACT-MCCTA_185
A-EACT-MCCAA_223
SL6-MCAC_087
A-EAAC-MCCATC_118
A-EACT-MCCAA_228
A-EAAC-MCCTA_065
A-EAGC-MCTC_324
SL4-MCCG_317
A-EAGC-MCTC_060
A-EAGC-MCTG_070
A-EAAG-MCCTA_066
A-EACT-MCCAA_206
SL6-MCTT_085p
A-EACG-MCCGC_056
A-EACG-MCCAC_123
A-EACG-MCTG_275
A-EACG-MCCAA_204p
A-EAGC-MCTC_138
A-EACG-MCAT_052p
A-EAGC-MCTC_385p
SL3-MCCG_416p
A-EAAC-MCCTA_107
SL4-MCAC_082
A-EAAC-MCCAC_228
A-EACG-MCCTA_238p
A-EAAG-MCCACC_098
SL4-MCTT_060
A-EACG-MCCTA_286
A-EACA-MCTA_154
A-EACG-MCCAA_126
A-EACG-MCCTA_128

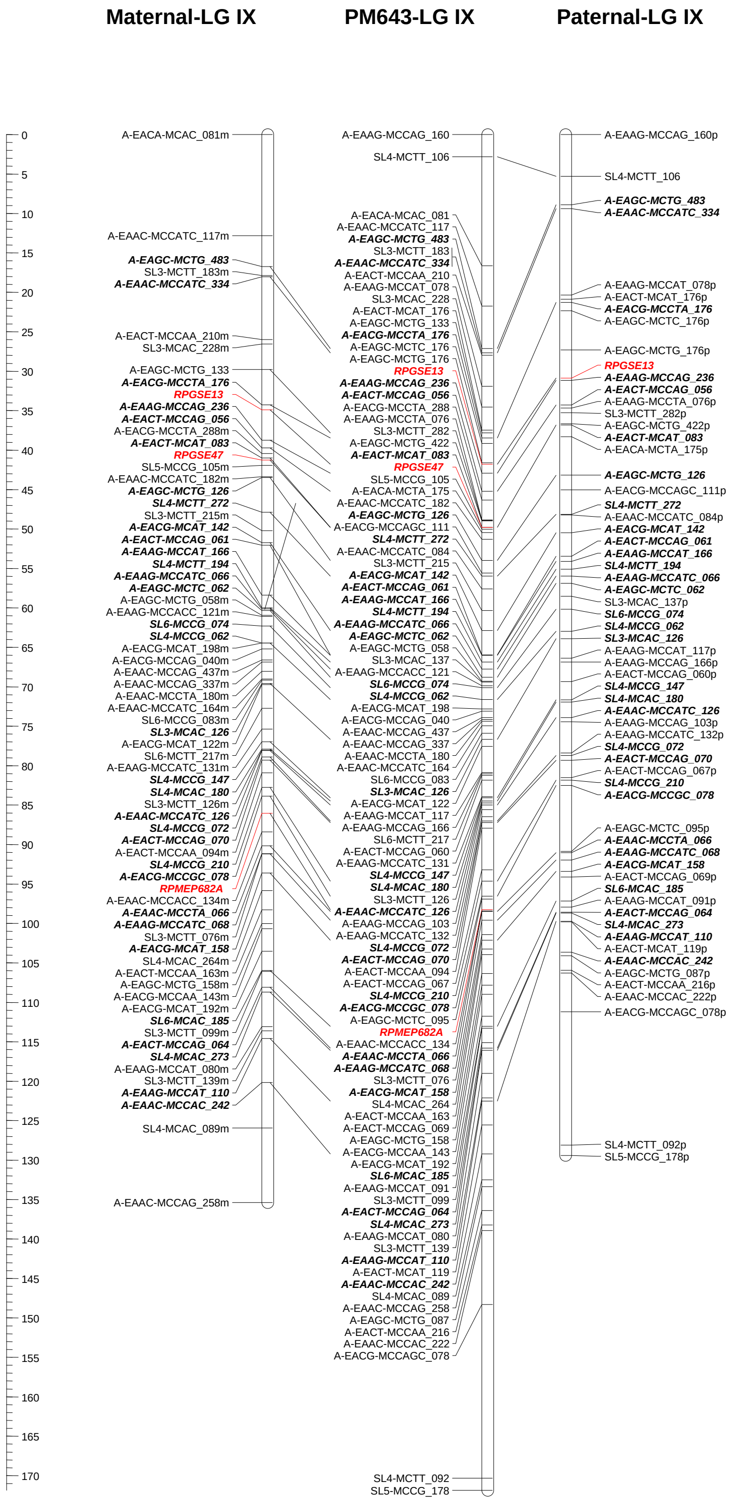

Maternal-LG IX
PM643-LG IX
Paternal-LG IX
0
5
10
15
20
25
30
35
40
45
50
55
60
65
70
75
80
85
90
95
100
105
110
115
120
125
130
135
140
145
150
155
160
165
170
A-EACA-MCAC_081m
A-EAAC-MCCATC_117m
A-EAGC-MCTG_483
SL3-MCTT_183m
A-EAAC-MCCATC_334
A-EACT-MCCAA_210m
SL3-MCAC_228m
A-EAGC-MCTG_133
A-EACG-MCCTA_176
RPGSE13
A-EAAG-MCCAG_236
A-EACT-MCCAG_056
A-EACG-MCCTA_288m
A-EACT-MCAT_083
RPGSE47
SL5-MCCG_105m
A-EAAC-MCCATC_182m
A-EAGC-MCTG_126
SL4-MCTT_272
SL3-MCTT_215m
A-EACG-MCAT_142
A-EACT-MCCAG_061
A-EAAG-MCCAT_166
SL4-MCTT_194
A-EAAG-MCCATC_066
A-EAGC-MCTC_062
A-EAGC-MCTG_058m
A-EAAG-MCCACC_121m
SL6-MCCG_074
SL4-MCCG_062
A-EACG-MCAT_198m
A-EACG-MCCAG_040m
A-EAAC-MCCAG_437m
A-EAAC-MCCAG_337m
A-EAAC-MCCTA_180m
A-EAAC-MCCATC_164m
SL6-MCCG_083m
SL3-MCAC_126
A-EACG-MCAT_122m
SL6-MCTT_217m
A-EAAG-MCCATC_131m
SL4-MCCG_147
SL4-MCAC_180
SL3-MCTT_126m
A-EAAC-MCCATC_126
SL4-MCCG_072
A-EACT-MCCAG_070
A-EACT-MCCAA_094m
SL4-MCCG_210
A-EACG-MCCGC_078
RPMEP682A
A-EAAC-MCCACC_134m
A-EAAC-MCCTA_066
A-EAAG-MCCATC_068
SL3-MCTT_076m
A-EACG-MCAT_158
SL4-MCAC_264m
A-EACT-MCCAA_163m
A-EAGC-MCTG_158m
A-EACG-MCCAA_143m
A-EACG-MCAT_192m
SL6-MCAC_185
SL3-MCTT_099m
A-EACT-MCCAG_064
SL4-MCAC_273
A-EAAG-MCCAT_080m
SL3-MCTT_139m
A-EAAG-MCCAT_110
A-EAAC-MCCAC_242
SL4-MCAC_089m
A-EAAC-MCCAG_258m
A-EAAG-MCCAG_160
SL4-MCTT_106
A-EACA-MCAC_081
A-EAAC-MCCATC_117
A-EAGC-MCTG_483
SL3-MCTT_183
A-EAAC-MCCATC_334
A-EACT-MCCAA_210
A-EAAG-MCCAT_078
SL3-MCAC_228
A-EACT-MCAT_176
A-EAGC-MCTG_133
A-EACG-MCCTA_176
A-EAGC-MCTG_176
A-EAGC-MCTG_176
RPGSE13
A-EAAG-MCCAG_236
A-EACT-MCCAG_056
A-EACG-MCCTA_288
A-EAAG-MCCTA_076
SL3-MCTT_282
A-EAGC-MCTG_422
A-EACT-MCAT_083
RPGSE47
SL5-MCCG_105
A-EACA-MCTA_175
A-EAAC-MCCATC_182
A-EAGC-MCTG_126
A-EACG-MCCAGC_111
SL4-MCTT_272
A-EAAC-MCCATC_084
SL3-MCTT_215
A-EACG-MCAT_142
A-EACT-MCCAG_061
A-EAAG-MCCAT_166
SL4-MCTT_194
A-EAAG-MCCATC_066
A-EAGC-MCTC_062
A-EAGC-MCTG_058
SL3-MCAC_137
A-EAAG-MCCACC_121
SL6-MCCG_074
SL4-MCCG_062
A-EACG-MCAT_198
A-EACG-MCCAG_040
A-EAAC-MCCAG_437
A-EAAC-MCCAG_337
A-EAAC-MCCTA_180
A-EAAC-MCCATC_164
SL6-MCCG_083
SL3-MCAC_126
A-EACG-MCAT_122
A-EAAG-MCCAT_117
A-EAAG-MCCAG_166
SL6-MCTT_217
A-EACT-MCCAG_060
A-EAAG-MCCACC_131
SL4-MCCG_147
SL4-MCAC_180
SL3-MCTT_126
A-EAAC-MCCATC_126
A-EAAG-MCCAG_103
A-EAAG-MCCATC_132
SL4-MCCG_072
A-EACT-MCCAG_070
A-EACT-MCCAA_094
A-EACT-MCCAG_067
SL4-MCCG_210
A-EACG-MCCGC_078
A-EAGC-MCTC_095
RPMEP682A
A-EAAC-MCCACC_134
A-EAAC-MCCTA_066
A-EAAG-MCCATC_068
SL3-MCTT_076
A-EACG-MCAT_158
SL4-MCAC_264
A-EACT-MCCAA_163
A-EACT-MCCAG_069
A-EAGC-MCTG_158
A-EACG-MCCAA_143
A-EACG-MCAT_192
SL6-MCAC_185
A-EAAG-MCCAT_091
SL3-MCTT_099
A-EACT-MCCAG_064
SL4-MCAC_273
A-EAAG-MCCAT_080
SL3-MCTT_139
A-EAAG-MCCAT_110
A-EACT-MCAT_119
A-EAAC-MCCAC_242
SL4-MCAC_089
A-EAAC-MCCAG_258
A-EAGC-MCTG_087
A-EACT-MCCAA_216
A-EAAC-MCCAC_222
A-EACG-MCCAGC_078
SL4-MCTT_092
SL5-MCCG_178
A-EAAG-MCCAG_160p
SL4-MCTT_106
A-EAGC-MCTG_483
A-EAAC-MCCATC_334
A-EAAG-MCCAT_078p
A-EACT-MCAT_176p
A-EACG-MCCTA_176
A-EAGC-MCTC_176p
A-EAGC-MCTG_176p
RPGSE13
A-EAAG-MCCAG_236
A-EACT-MCCAG_056
A-EAAG-MCCTA_076p
SL3-MCTT_282p
A-EAGC-MCTG_422p
A-EACT-MCAT_083
A-EACA-MCTA_175p
A-EAGC-MCTG_126
A-EACG-MCCAGC_111p
SL4-MCTT_272
A-EAAC-MCCATC_084p
A-EACG-MCAT_142
A-EACT-MCCAG_061
A-EAAG-MCCAT_166
SL4-MCTT_194
A-EAAG-MCCATC_066
A-EAGC-MCTC_062
SL3-MCAC_137p
SL6-MCCG_074
SL4-MCCG_062
SL3-MCAC_126
A-EAAG-MCCAT_117p
A-EAAG-MCCAG_166p
A-EACT-MCCAG_060p
SL4-MCCG_147
SL4-MCAC_180
A-EAAC-MCCATC_126
A-EAAG-MCCAG_103p
A-EAAG-MCCATC_132p
SL4-MCCG_072
A-EACT-MCCAG_070
A-EACT-MCCAG_067p
SL4-MCCG_210
A-EACG-MCCGC_078
A-EAGC-MCTC_095p
A-EAAC-MCCTA_066
A-EAAG-MCCATC_068
A-EACG-MCAT_158
A-EACT-MCCAG_069p
SL6-MCAC_185
A-EAAG-MCCAT_091p
A-EACT-MCCAG_064
SL4-MCAC_273
A-EAAG-MCCAT_110
A-EACT-MCAT_119p
A-EAAC-MCCAC_242
A-EAGC-MCTG_087p
A-EACT-MCCAA_216p
A-EAAC-MCCAC_222p
A-EACG-MCCAGC_078p
SL4-MCTT_092p
SL5-MCCG_178p

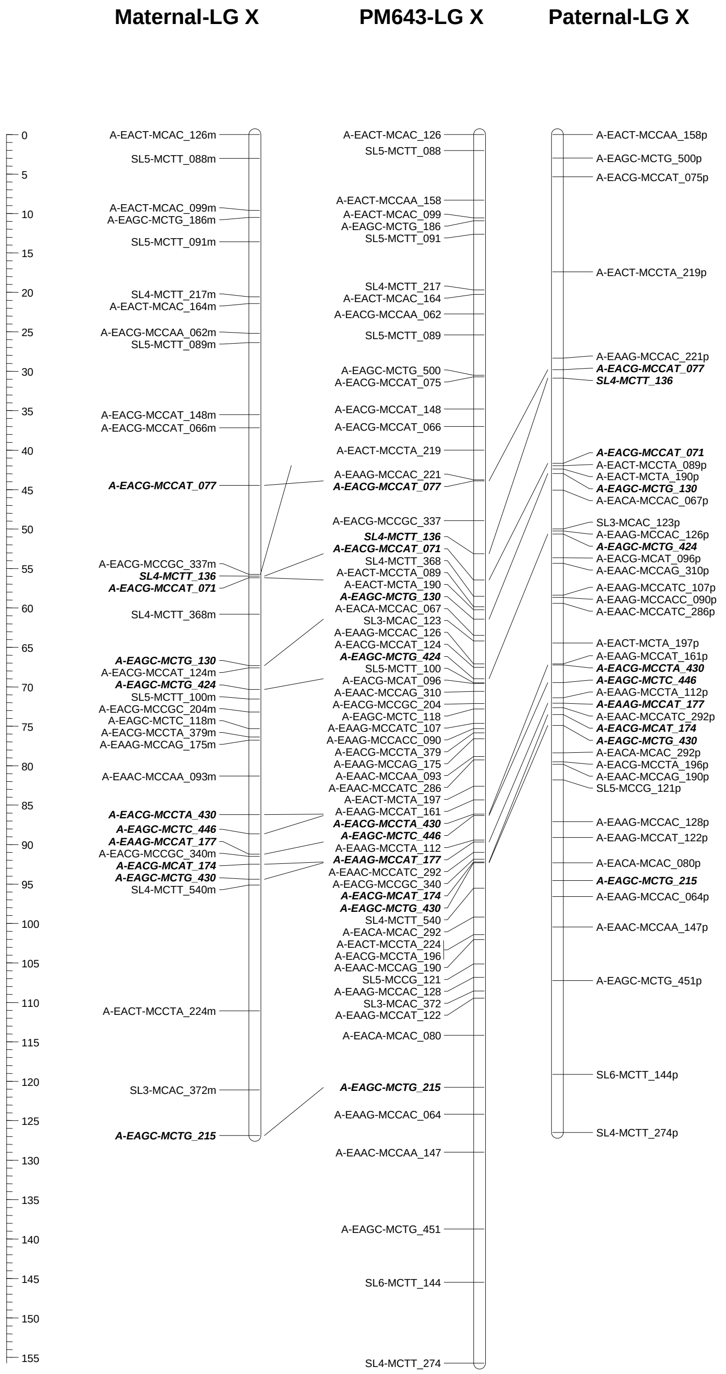

Maternal-LG X
PM643-LG X
Paternal-LG X
A-EACT-MCAC_126m
SL5-MCTT_088m
A-EACT-MCAC_099m
A-EAGC-MCTG_186m
SL5-MCTT_091m
SL4-MCTT_217m
A-EACT-MCAC_164m
A-EACG-MCCAA_062m
SL5-MCTT_089m
A-EACG-MCCAT_148m
A-EACG-MCCAT_066m
A-EACG-MCCAT_077
A-EACG-MCCGC_337m
SL4-MCTT_136
A-EACG-MCCAT_071
SL4-MCTT_368m
A-EAGC-MCTG_130
A-EACG-MCCAT_124m
A-EAGC-MCTG_424
SL5-MCTT_100m
A-EACG-MCCGC_204m
A-EAGC-MCTC_118m
A-EACG-MCCTA_379m
A-EAAG-MCCAG_175m
A-EAAC-MCCAA_093m
A-EACG-MCCTA_430
A-EAGC-MCTC_446
A-EAAG-MCCAT_177
A-EACG-MCCGC_340m
A-EACG-MCAT_174
A-EAGC-MCTG_430
SL4-MCTT_540m
A-EACT-MCCTA_224m
SL3-MCAC_372m
A-EAGC-MCTG_215
A-EACT-MCAC_126
SL5-MCTT_088
A-EACT-MCCAA_158
A-EACT-MCAC_099
A-EAGC-MCTG_186
SL5-MCTT_091
SL4-MCTT_217
A-EACT-MCAC_164
A-EACG-MCCAA_062
SL5-MCTT_089
A-EAGC-MCTG_500
A-EACG-MCCAT_075
A-EACG-MCCAT_148
A-EACG-MCCAT_066
A-EACT-MCCTA_219
A-EAAG-MCCAC_221
A-EACG-MCCAT_077
A-EACG-MCCGC_337
SL4-MCTT_136
A-EACG-MCCAT_071
SL4-MCTT_368
A-EACT-MCCTA_089
A-EACT-MCTA_190
A-EAGC-MCTG_130
A-EACA-MCCAC_067
SL3-MCAC_123
A-EAAG-MCCAC_126
A-EACG-MCCAT_124
A-EAGC-MCTG_424
SL5-MCTT_100
A-EACG-MCAT_096
A-EAAC-MCCAG_310
A-EACG-MCCGC_204
A-EAGC-MCTC_118
A-EAAG-MCCATC_107
A-EAAG-MCCACC_090
A-EACG-MCCTA_379
A-EAAG-MCCAG_175
A-EAAC-MCCAA_093
A-EAAC-MCCATC_286
A-EACT-MCTA_197
A-EAAG-MCCAT_161
A-EACG-MCCTA_430
A-EAGC-MCTC_446
A-EAAG-MCCTA_112
A-EAAG-MCCAT_177
A-EAAC-MCCATC_292
A-EACG-MCCGC_340
A-EACG-MCAT_174
A-EAGC-MCTG_430
SL4-MCTT_540
A-EACA-MCAC_292
A-EACT-MCCTA_224
A-EACG-MCCTA_196
A-EAAC-MCCAG_190
SL5-MCCG_121
A-EAAG-MCCAC_128
SL3-MCAC_372
A-EAAG-MCCAT_122
A-EACA-MCAC_080
A-EAGC-MCTG_215
A-EAAG-MCCAC_064
A-EAAC-MCCAA_147
A-EAGC-MCTG_451
SL6-MCTT_144
SL4-MCTT_274
A-EACT-MCCAA_158p
A-EAGC-MCTG_500p
A-EACG-MCCAT_075p
A-EACT-MCCTA_219p
A-EAAG-MCCAC_221p
A-EACG-MCCAT_077
SL4-MCTT_136
A-EACG-MCCAT_071
A-EACT-MCCTA_089p
A-EACT-MCTA_190p
A-EAGC-MCTG_130
A-EACA-MCCAC_067p
SL3-MCAC_123p
A-EAAG-MCCAC_126p
A-EAGC-MCTG_424
A-EACG-MCAT_096p
A-EAAC-MCCAG_310p
A-EAAG-MCCATC_107p
A-EAAG-MCCACC_090p
A-EAAC-MCCATC_286p
A-EACT-MCTA_197p
A-EAAG-MCCAT_161p
A-EACG-MCCTA_430
A-EAGC-MCTC_446
A-EAAG-MCCTA_112p
A-EAAG-MCCAT_177
A-EAAC-MCCATC_292p
A-EACG-MCAT_174
A-EAGC-MCTG_430
A-EACA-MCAC_292p
A-EACG-MCCTA_196p
A-EAAC-MCCAG_190p
SL5-MCCG_121p
A-EAAG-MCCAC_128p
A-EAAG-MCCAT_122p
A-EACA-MCAC_080p
A-EAGC-MCTG_215
A-EAAG-MCCAC_064p
A-EAAC-MCCAA_147p
A-EAGC-MCTG_451p
SL6-MCTT_144p
SL4-MCTT_274p
0
5
10
15
20
25
30
35
40
45
50
55
60
65
70
75
80
85
90
95
100
105
110
115
120
125
130
135
140
145
150
155

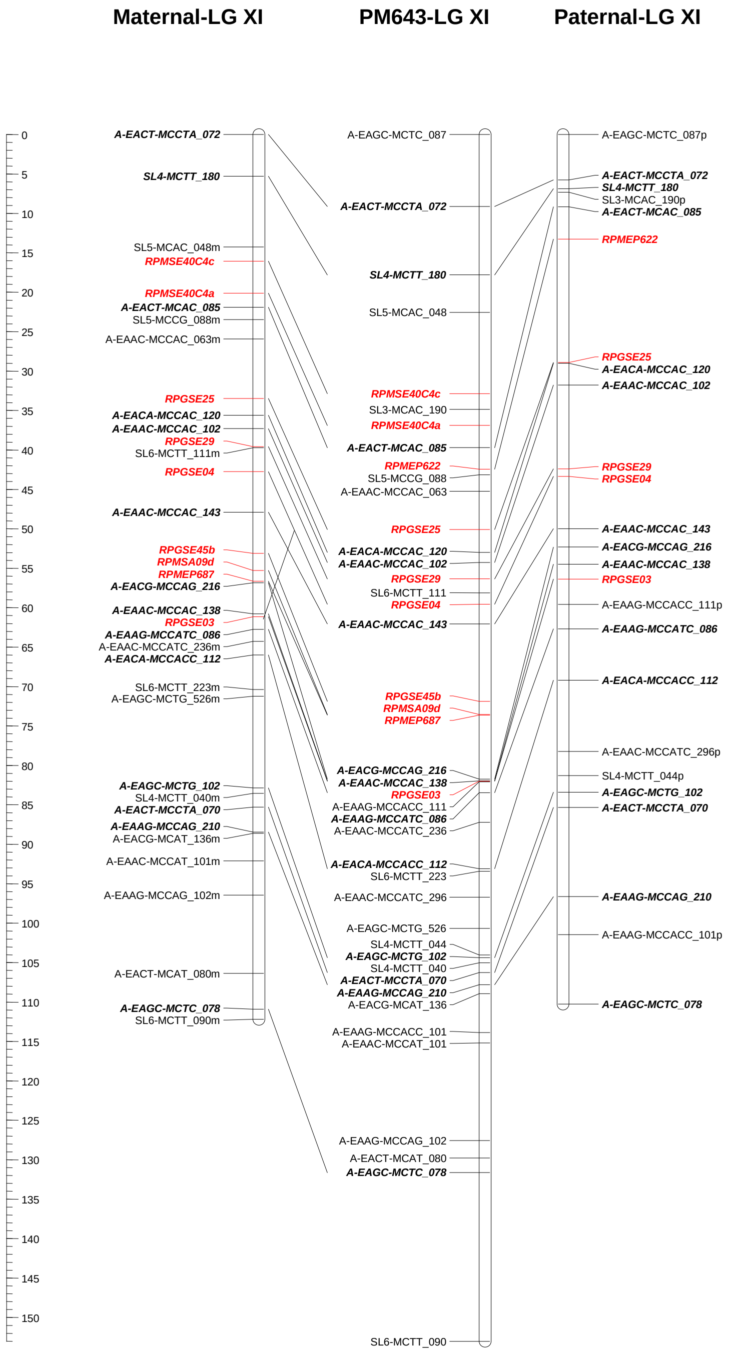

Maternal-LG XI
PM643-LG XI
Paternal-LG XI
0
5
10
15
20
25
30
35
40
45
50
55
60
65
70
75
80
85
90
95
100
105
110
115
120
125
130
135
140
145
150
A-EACT-MCCTA_072
SL4-MCTT_180
SL5-MCAC_048m
RPMSE40C4c
RPMSE40C4a
A-EACT-MCAC_085
SL5-MCCG_088m
A-EAAC-MCCAC_063m
RPGSE25
A-EACA-MCCAC_120
A-EAAC-MCCAC_102
RPGSE29
SL6-MCTT_111m
RPGSE04
A-EAAC-MCCAC_143
RPGSE45b
RPMSA09d
RPMEP687
A-EACG-MCCAG_216
A-EAAC-MCCAC_138
RPGSE03
A-EAAG-MCCATC_086
A-EAAC-MCCATC_236m
A-EACA-MCCACC_112
SL6-MCTT_223m
A-EAGC-MCTG_526m
A-EAGC-MCTG_102
SL4-MCTT_040m
A-EACT-MCCTA_070
A-EAAG-MCCAG_210
A-EACG-MCAT_136m
A-EAAC-MCCAT_101m
A-EAAG-MCCAG_102m
A-EACT-MCAT_080m
A-EAGC-MCTC_078
SL6-MCTT_090m
A-EAGC-MCTC_087
A-EACT-MCCTA_072
SL4-MCTT_180
SL5-MCAC_048
RPMSE40C4c
SL3-MCAC_190
RPMSE40C4a
A-EACT-MCAC_085
RPMEP622
SL5-MCCG_088
A-EAAC-MCCAC_063
RPGSE25
A-EACA-MCCAC_120
A-EAAC-MCCAC_102
RPGSE29
SL6-MCTT_111
RPGSE04
A-EAAC-MCCAC_143
RPGSE45b
RPMSA09d
RPMEP687
A-EACG-MCCAG_216
A-EAAC-MCCAC_138
RPGSE03
A-EAAG-MCCACC_111
A-EAAG-MCCATC_086
A-EAAC-MCCATC_236
A-EACA-MCCACC_112
SL6-MCTT_223
A-EAAC-MCCATC_296
A-EAGC-MCTG_526
SL4-MCTT_044
A-EAGC-MCTG_102
SL4-MCTT_040
A-EACT-MCCTA_070
A-EAAG-MCCAG_210
A-EACG-MCAT_136
A-EAAG-MCCACC_101
A-EAAC-MCCAT_101
A-EAAG-MCCAG_102
A-EACT-MCAT_080
A-EAGC-MCTC_078
SL6-MCTT_090
A-EAGC-MCTC_087p
A-EACT-MCCTA_072
SL4-MCTT_180
SL3-MCAC_190p
A-EACT-MCAC_085
RPMEP622
RPGSE25
A-EACA-MCCAC_120
A-EAAC-MCCAC_102
RPGSE29
RPGSE04
A-EAAC-MCCAC_143
A-EACG-MCCAG_216
A-EAAC-MCCAC_138
RPGSE03
A-EAAG-MCCACC_111p
A-EAAG-MCCATC_086
A-EACA-MCCACC_112
A-EAAC-MCCATC_296p
SL4-MCTT_044p
A-EAGC-MCTG_102
A-EACT-MCCTA_070
A-EAAG-MCCAG_210
A-EAAG-MCCACC_101p
A-EAGC-MCTC_078

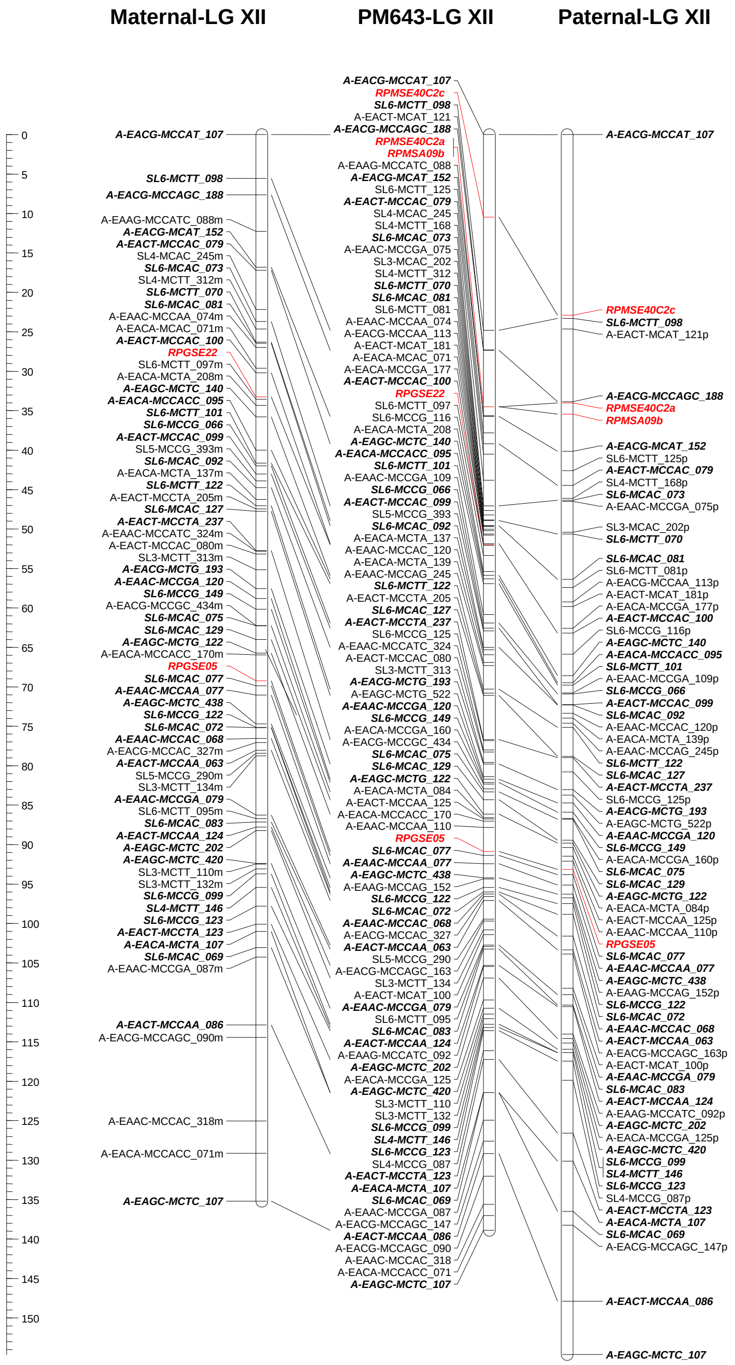
Maternal-LG XII
PM643-LG XII
Paternal-LG XII
0
5
10
15
20
25
30
35
40
45
50
55
60
65
70
75
80
85
90
95
100
105
110
115
120
125
130
135
140
145
150
A-EACG-MCCAT_107
SL6-MCTT_098
A-EACG-MCCAGC_188
A-EAAG-MCCATC_088m
A-EACG-MCAT_152
A-EACT-MCCAC_079
SL4-MCAC_245m
SL6-MCAC_073
SL4-MCTT_312m
SL6-MCTT_070
SL6-MCAC_081
A-EAAC-MCCAA_074m
A-EACA-MCAC_071m
A-EACT-MCCAC_100
RPGSE22
SL6-MCTT_097m
A-EACA-MCTA_208m
A-EAGC-MCTC_140
A-EACA-MCCACC_095
SL6-MCTT_101
SL6-MCCG_066
A-EACT-MCCAC_099
SL5-MCCG_393m
SL6-MCAC_092
A-EACA-MCTA_137m
SL6-MCTT_122
A-EACT-MCCTA_205m
SL6-MCAC_127
A-EACT-MCCTA_237
A-EAAC-MCCATC_324m
A-EACT-MCCAC_080m
SL3-MCTT_313m
A-EACG-MCTG_193
A-EAAC-MCCGA_120
SL6-MCCG_149
A-EACG-MCCGC_434m
SL6-MCAC_075
SL6-MCAC_129
A-EAGC-MCTG_122
A-EACA-MCCACC_170m
RPGSE05
SL6-MCAC_077
A-EAAC-MCCAA_077
A-EAGC-MCTC_438
SL6-MCCG_122
SL6-MCAC_072
A-EAAC-MCCAC_068
A-EACG-MCCAC_327m
A-EACT-MCCAA_063
SL5-MCCG_290m
SL3-MCTT_134m
A-EAAC-MCCGA_079
SL6-MCTT_095m
SL6-MCAC_083
A-EACT-MCCAA_124
A-EAGC-MCTC_202
A-EAGC-MCTC_420
SL3-MCTT_110m
SL3-MCTT_132m
SL6-MCCG_099
SL4-MCTT_146
SL6-MCCG_123
A-EACT-MCCTA_123
A-EACA-MCTA_107
SL6-MCAC_069
A-EAAC-MCCGA_087m
A-EACT-MCCAA_086
A-EACG-MCCAGC_090m
A-EAAC-MCCAC_318m
A-EACA-MCCACC_071m
A-EAGC-MCTC_107
A-EACG-MCCAT_107
RPMSE40C2c
SL6-MCTT_098
A-EACT-MCAT_121
A-EACG-MCCAGC_188
RPMSE40C2a
RPMSA09b
A-EAAG-MCCATC_088
A-EACG-MCAT_152
SL6-MCTT_125
A-EACT-MCCAC_079
SL4-MCAC_245
SL4-MCTT_168
SL6-MCAC_073
A-EAAC-MCCGA_075
SL3-MCAC_202
SL4-MCTT_312
SL6-MCTT_070
SL6-MCAC_081
SL6-MCTT_081
A-EAAC-MCCAA_074
A-EACG-MCCAA_113
A-EACT-MCAT_181
A-EACA-MCAC_071
A-EACA-MCCGA_177
A-EACT-MCCAC_100
RPGSE22
SL6-MCTT_097
SL6-MCCG_116
A-EACA-MCTA_208
A-EAGC-MCTC_140
A-EACA-MCCACC_095
SL6-MCTT_101
A-EAAC-MCCGA_109
SL6-MCCG_066
A-EACT-MCCAC_099
SL5-MCCG_393
SL6-MCAC_092
A-EACA-MCTA_137
A-EAAC-MCCAC_120
A-EACA-MCTA_139
A-EAAC-MCCAG_245
SL6-MCTT_122
A-EACT-MCCTA_205
SL6-MCAC_127
A-EACT-MCCTA_237
SL6-MCCG_125
A-EAAC-MCCATC_324
A-EACT-MCCAC_080
SL3-MCTT_313
A-EACG-MCTG_193
A-EAGC-MCTG_522
A-EAAC-MCCGA_120
SL6-MCCG_149
A-EACA-MCCGA_160
A-EACG-MCCGC_434
SL6-MCAC_075
SL6-MCAC_129
A-EAGC-MCTG_122
A-EACA-MCTA_084
A-EACT-MCCAA_125
A-EACA-MCCACC_170
A-EAAC-MCCAA_110
RPGSE05
SL6-MCAC_077
A-EAAC-MCCAA_077
A-EAGC-MCTC_438
A-EAAG-MCCAG_152
SL6-MCCG_122
SL6-MCAC_072
A-EAAC-MCCAC_068
A-EACG-MCCAC_327
A-EACT-MCCAA_063
SL5-MCCG_290
A-EACG-MCCAGC_163
SL3-MCTT_134
A-EACT-MCAT_100
A-EAAC-MCCGA_079
SL6-MCTT_095
SL6-MCAC_083
A-EACT-MCCAA_124
A-EAAG-MCCATC_092
A-EAGC-MCTC_202
A-EACA-MCCGA_125
A-EAGC-MCTC_420
SL3-MCTT_110
SL3-MCTT_132
SL6-MCCG_099
SL4-MCTT_146
SL6-MCCG_123
SL4-MCCG_087
A-EACT-MCCTA_123
A-EACA-MCTA_107
SL6-MCAC_069
A-EAAC-MCCGA_087
A-EACG-MCCAGC_147
A-EACT-MCCAA_086
A-EACG-MCCAGC_090
A-EAAC-MCCAC_318
A-EACA-MCCACC_071
A-EAGC-MCTC_107
A-EACG-MCCAT_107
RPMSE40C2c
SL6-MCTT_098
A-EACT-MCAT_121p
A-EACG-MCCAGC_188
RPMSE40C2a
RPMSA09b
A-EACG-MCAT_152
SL6-MCTT_125p
A-EACT-MCCAC_079
SL4-MCTT_168p
SL6-MCAC_073
A-EAAC-MCCGA_075p
SL3-MCAC_202p
SL6-MCTT_070
SL6-MCAC_081
SL6-MCTT_081p
A-EACG-MCCAA_113p
A-EACT-MCAT_181p
A-EACA-MCCGA_177p
A-EACT-MCCAC_100
SL6-MCCG_116p
A-EAGC-MCTC_140
A-EACA-MCCACC_095
SL6-MCTT_101
A-EAAC-MCCGA_109p
SL6-MCCG_066
A-EACT-MCCAC_099
SL6-MCAC_092
A-EAAC-MCCAC_120p
A-EACA-MCTA_139p
A-EAAC-MCCAG_245p
SL6-MCTT_122
SL6-MCAC_127
A-EACT-MCCTA_237
SL6-MCCG_125p
A-EACG-MCTG_193
A-EAGC-MCTG_522p
A-EAAC-MCCGA_120
SL6-MCCG_149
A-EACA-MCCGA_160p
SL6-MCAC_075
SL6-MCAC_129
A-EAGC-MCTG_122
A-EACA-MCTA_084p
A-EACT-MCCAA_125p
A-EAAC-MCCAA_110p
RPGSE05
SL6-MCAC_077
A-EAAC-MCCAA_077
A-EAGC-MCTC_438
A-EAAG-MCCAG_152p
SL6-MCCG_122
SL6-MCAC_072
A-EAAC-MCCAC_068
A-EACT-MCCAA_063
A-EACG-MCCAGC_163p
A-EACT-MCAT_100p
A-EAAC-MCCGA_079
SL6-MCAC_083
A-EACT-MCCAA_124
A-EAAG-MCCATC_092p
A-EAGC-MCTC_202
A-EACA-MCCGA_125p
A-EAGC-MCTC_420
SL6-MCCG_099
SL4-MCTT_146
SL6-MCCG_123
SL4-MCCG_087p
A-EACT-MCCTA_123
A-EACA-MCTA_107
SL6-MCAC_069
A-EACG-MCCAGC_147p
A-EACT-MCCAA_086
A-EAGC-MCTC_107
