# Supplementary material for: Near-saturated and complete genetic linkage map of black spruce (Picea mariana)
Source: BMC Genomics. 2010 Sep 24;11:515. doi: 10.1186/1471-2164-11-515 (PMC2997009; doi:10.1186/1471-2164-11-515)
Supplement: Additional file 2 — Table S1 Expressed sequence tag polymorphism (ESTP) and EST-based microsatellite (SSR) markers developed from black spruce ESTs and mapped on the black spruce genetic map in this study. [file 1471-2164-11-515-S2.DOC]

**Additional File 2**

**Table S1 Expressed sequence tag polymorphic (ESTP) and EST-based microsatellite (SSR) markers developed from black spruce ESTs and mapped on the black spruce genetic map in this study.**

| **Name of the ESTP or SSR locus** | **Forward (F) and reverse (R) primer sequences (5' - 3')** | **Annealing**  **temperature (°C)** |
| --- | --- | --- |
|  |  |  |
| **ESTP Markers** | | |
| RPMEP 622 | F-CACGGACGATTCCACTGTC  R-CGGCATCAGCATTAGCCCGT | 60 |
| RPMEP682A | F-CGGTCTCTCCTTCGACTCAC  R-CAGAAAAGATCTTCAGCCCC | 55 |
| RPMEP682B | F-CGGTCTCTCCTTCGACTCAC  R-CAGAAAAGATCTTCAGCCCC | 55 |
| RPMEP638 | F-AGATCTCAGAGTCTGTGCTTTGC  R-ACAATCCTGCCAAGTCCCC | 55 |
| RPMEP687 | F-CAGAAATGGCAAGAAAGGGA  R-CTATATCACAAAGAAAAATCTAGC | 60 |
| **EST Microsatellite Markers** | | |
| RPMSE40C2a | F: GAAATGAATGAGTGAATTTAAGGC  R: CCAAATATTATCCCGACAATCC | 60 |
| RPMSE40C2c | F: GAAATGAATGAGTGAATTTAAGGC  R: CCAAATATTATCCCGACAATCC | 60 |
| RPMSE40C2d | F: GAAATGAATGAGTGAATTTAAGGC  R: CCAAATATTATCCCGACAATCC | 60 |
| RPMSE40C4a | F: GAATCACTGGAAGGGCCTAA  R: GGATTTGCATTGCCTTTTGT | 60 |
| RPMSE40C4c | F: GAATCACTGGAAGGGCCTAA  R: GGATTTGCATTGCCTTTTGT | 60 |
| RPMSE40C12 | F: CAATGGTGCATAGGCATCTC  R: CAACAAAAGGCCTCAGCAAT | 60 |
